# Daqinjiao Decoction Ameliorates CSVD via RXR‐γ/PPAR‐γ/VEGF‐α Pathway: Insights From Transcriptome Sequencing and Network Pharmacology

**DOI:** 10.1111/jcmm.70712

**Published:** 2025-07-19

**Authors:** Mengna Lv, Xiaolu Yang, Xiaolu Shi, Shengxuan Cao, Wenjie Li, Mingmei Zhou, Xiaojun Gou, Ying Huang

**Affiliations:** ^1^ Institute of Interdisciplinary Integrative Medicine Research Shanghai University of Traditional Chinese Medicine Shanghai China; ^2^ Experimental Research Center Beijing Key Laboratory of Research of Chinese Medicine on Prevention and Treatment for Major Diseases, China Academy of Chinese Medical Sciences Beijing China; ^3^ Central Laboratory, Baoshan District Hospital of Integrated Traditional Chinese and Western Medicine of Shanghai Shanghai University of Traditional Chinese Medicine Shanghai China

**Keywords:** cerebral small vessel disease (CSVD), Daqinjiao decoction (DQJD), network pharmacology, RXR‐γ/PPAR‐γ/VEGF‐α pathway, transcriptome sequencing

## Abstract

Daqinjiao decoction (DQJD), an ancient traditional Chinese medicine formula, is clinically used in the treatment of cerebral small vessel disease (CSVD). However, the underlying molecular mechanisms by which DQJD exerts its therapeutic effects on CSVD remain elusive. A Sprague Dawley rat model of chronic cerebral hypoperfusion (CCH) was established using bilateral common carotid artery occlusion (BCCAO) surgery. The effects of DQJD administered via intragastric gavage (i.g.) were evaluated by magnetic resonance imaging (MRI), haematoxylin eosin (HE) staining, and transmission electron microscopy. A combined strategy of transcriptomics and network pharmacology was innovatively applied to study the active ingredients, gene targets, and mechanisms of DQJD in treating CSVD. Molecular docking, real‐time quantitative polymerase chain reaction (RT‐qPCR), and Western blot analysis were applied to confirm the above results further. DQJD improved the pathological damage in cortical and hippocampal tissue by reducing the release of inflammatory factors in CCH rats. RNA‐seq technology identified 46 DEGs between DQJD treatment and the model group. Network pharmacology and pathway analysis of DEGs revealed that the PPAR/VEGF signalling pathway was predicted to be significantly affected. Consistently, validation experiments confirmed that activation of the RXR‐γ/PPAR‐γ/VEGF‐α signalling pathway represents a key mechanism underlying DQJD's therapeutic effects against CSVD. DQJD dampened inflammation and ameliorated pathological damage in the hippocampus and cortex, and these beneficial effects were associated with neurovascular protection via activating the RXR‐γ/PPAR‐γ/VEGF‐α pathway.

**Abbreviations**
ANOVA: one‐way analysis of variance
BBB: blood–brain barrier
BCCAO: bilateral common carotid artery occlusion
CBF: cerebral blood flow
CCH: chronic cerebral hypoperfusion
COX‐2: cyclooxygenase‐2
CSVD: cerebral small vessel disease
DEGs: differentially expressed genes
DQJD: daqinjiao decoction
DWI: diffusion‐weighted imaging
GAPDH: glyceraldehyde‐3‐phosphate dehydrogenase
GO: gene ontology
HE: haematoxylin and eosin
HIF‐1α: hypoxia‐inducible factor
ICAM‐1: intercellular adhesion molecule 1
IL‐1β: interleukin 1 beta
KEGG: kyoto encyclopedia of genes and genomes
MRI: magnetic resonance imaging
PCA: principal component analysis
PGC‐1α: peroxisome proliferator activated receptor gamma coactivator‐1α
PPAR‐γ: peroxisome proliferator activated receptor gamma
RNA‐seq: RNA sequencing
RT‐qPCR: real‐time quantitative polymerase chain reaction
RXRG: retinoid × receptor gamma
TNF‐α: tumour necrosis factor alpha
VEGF‐α: vascular endothelial growth factor

## 1 Introduction

Cerebral small vessel disease (CSVD), a common symptom of aging, refers to pathological processes of the small vessels in the brain, manifesting as stroke or gait impairment clinically [1]. Its neuroimaging features include subcortical infarcts, lacunae, white matter high signal, perivascular gap microhemorrhages and cerebral atrophy [2]. Chronic cerebral hypoperfusion (CCH) has been considered the main cause of CSVD. Currently, there is a broad consensus regarding the causes of CCH, which include neurovascular imbalance after continuous reduction of cerebral blood flow (CBF), oxidative stress damage, white matter damage and blood–brain barrier (BBB) damage, neuroinflammatory response, lipid metabolism disorder, and changes in growth factors and so forth. These are all related to the ultimate neuronal death, and the loss of neuronal cells in the cortex and hippocampus is one of the focal points of CCH‐induced CSVD.

CCH reduces blood flow and causes inflammation, which are classical pathological features of CSVD and could be a major etiological factor of CSVD [3]. Inflammatory responses can be triggered by cytokines (e.g., tumour necrosis factor alpha (TNF‐α), interleukin 1 beta (IL‐1β) and cyclooxygenase‐2 (COX‐2)) and adhesion molecules (e.g., intercellular adhesion molecule 1 (ICAM‐1)) after CCH. These factors enhance the infiltration of leukocytes, macrophages, and reactive microglia in the ischaemic area, ultimately leading to delayed neuronal death [4].

Traditional Chinese medicine has a long history of treating CSVD and has exhibited unique efficacy [5]. Daqinjiao decoction (DQJD) is an ancient herbal formula and was originally documented in a medical work written by Wansu Liu (1100–1200) [6]. The obvious application value is dedicated to the treatment of progressive stroke. Contemporary pharmacological research has confirmed that DQJD significantly improves prothrombin time, reduces fibrinogen, and decreases platelet adhesion and aggregation rates in cerebral ischaemia [7]. In the clinic, DQJD significantly enhances cerebral circulation, promotes tissue perfusion, and expands cerebral arteries, lowering the toxicity of brain tissue damage caused by ischaemia and reperfusion. Additionally, it exerts antioxidant and anti‐inflammation effects, including preventing lipid peroxidation, enhancing the ability of the body to neutralise free radicals and reducing serum levels of inflammatory factors in patients with acute cerebral infarction [8].

Based on the ‘network target, multicomponent therapeutics’ strategy, network pharmacology describes the sophisticated connections between drugs, biological systems, and disorders from a holistic perspective [9]. The transcriptome sequencing technique is a powerful tool to analyse transcriptome using high‐throughput sequencing. It has the advantage of rapidly identifying the mRNA changes after drug treatment, which contributes to the comprehension and elucidation of the molecular mechanism of action [10]. In recent years, the research strategy integrating network pharmacology with transcriptomics has been recognised as a suitable method for exploring the mechanisms underlying the therapeutic effects of traditional Chinese medicine due to its both macroscopic and microscopic features. Integrating transcriptomic techniques, network pharmacology, and pharmacodynamic experiments, Wang et al. demonstrated that valerian essential oil improves insomnia symptoms and relieves anxiety via the serotonergic synapse pathway [11]. Transcriptome sequencing and network pharmacology‐based approach have been used to explore the biological pathways of Ji Chuan Jian in treating PD, with results indicating that the underlying mechanism may involve matrix metalloproteinases‐9, HIF‐1 signalling pathway and IL‐17 signalling pathway [12].

In the present study, the status of CBF and neuronal death in the brain of the CCH model was evaluated using magnetic resonance imaging (MRI) and haematoxylin and eosin (HE) staining to confirm the neuroprotective effect of DQJD in the progression of CSVD. Integrating network pharmacology with transcriptome sequencing analysis was conducted to explore the molecular mechanisms of DQJD against CSVD in rats. Real‐time quantitative polymerase chain reaction (RT‐qPCR) and the western blot assay were used to confirm the predicted mechanisms.

## 2 Materials and Methods

### 2.1 Materials

All herbs of DQJD were purchased from Beijing Tong Ren Tang Co. Ltd. (Table 1) and authenticated by Dr. Xirong He from the China Academy of Chinese Medical Sciences (Beijing, China). All herbs comply with the property regulations under the relevant items of the 2020 edition of the Chinese Pharmacopoeia. 2.5% glutaraldehyde (SBJ‐0639, Nanjing SenBeiJia Biological Technology Co. Ltd.), osmium tetroxide (18,456, Ted Pella Inc), acetone (10,000,418, Sinopharm Chemical Reagent Co. Ltd.), epoxy resin (02660, SPI), RIPA buffer (Lot. R0010) and PMSF solution (Lot. R0100) were procured from Beijing Solarbio Science & Technology Co. Ltd., China. VEGFA antibody (Lot.19003‐1‐AP), RXRG antibody (Lot.11129‐1‐AP), and PPAR antibody (Lot.16643‐1‐AP) were purchased from Proteintech (Rosemont, USA). GAPDH antibody (Lot.bs‐10900R) was purchased from Beijing Biosynthesis Biotechnology Co. Ltd. (Beijing, China). Rat ELISA kits of TNF‐α (Lot.EK382/3), COX‐2 (Lot.CSB‐E13399r) and ICAM‐1 (Lot.PI495) were purchased from Multi Sciences (Lianke) Biotech Co. Ltd. (Zhejiang, China), CUSABIO Co. Ltd. (Wuhan, China), and Beyotime Biotech Inc. (Shanghai, China), respectively.

**TABLE 1:** Components of DQJD.

Herbs (Chinese Pinyin)	Herbs (Latin name)	Herbs (English name)	Amount (g)
Qinjiao	*Gentiana macrophylla Pall*.	Gentianae Macrophyllae Radix	9
Gancao	*Glycyrrhiza uralensis Fisch*.	Glycyrrhizae Radix et Rhizoma	6
Chuanxiong	*Ligusticum chuanxiong Hort*.	Chuanxiong Rhizoma	6
Danggui	*Angelica sinensis (Oliv.) Diels*	Angelicae Sinensis Radix	6
Baishao	*Paeonia lactiflora Pall*.	Paeoniae Radix Alba	6
Shigao	—	Gypsum Fibrosum	6
Duhuo	*Angelica pubescens Maxim.f. biserrata Shan et Yuan*	Angelicae Pubescentis Radix	6
Qianghuo	*Notopterygium incisum Ting ex H. T. Chang*	Notopterygii Rhizoma et Radix	3
Fangfeng	*Saposhnikovia divaricata (Turcz.) Schischk*.	Saposhnikoviae Radix	3
Huangqin	*Scutellaria baicalensis Georgi*	Scutellariae Radix	3
Baizhi	*Angelica dahurica (Fisch.ex Hoffm.) Benth.et Hook.f*.	Angelicae Dahuricae Radix	3
Baizhu	*Atractylodes macrocephala Koidz*.	Atractylodis Macrocephalae Rhizoma	3
Shengdihuang	*Rehmannia glutinosa Libosch*.	Rehmanniae Radix	3
Shudihuang	*Rehmannia glutinosa Libosch*.	Rehmanniae Radix Praeparata	3
Fuling	*Poria cocos (Schw.) Wolf*	Poria	3
Xixin	*Asarum heterotropoides Fr. Schmidt var. mandshuricum (Maxim.) Kitag*.	Asari Radix et Rhizoma	1.5

The following instruments were used: Transmission electron microscopy (H7650, Hitachi Ltd.), automatic dehydrator (JJ‐2J, Wuhan Junjie Electronics Co. Ltd.), tissue embedding machine (JB‐P5, Wuhan Junjie Electronics Co. Ltd.), rotary microtome (RM2016, Leica Instruments Co. Ltd., Shanghai.), cryo‐embedding platform (JB‐L5, Wuhan Junjie Electronics Co. Ltd.), tissue flotation system (KD‐P, Zhejiang Kedi Instrument Co. Ltd.), drying oven (DHG‐9140A, Shanghai Huitai Instrument Co. Ltd.), microscopy slides (10212432C, CITOTEST Scientific, Jiangsu), upright optical microscope (Eclipse Ci, Nikon, Japan) and digital imaging system (DS‐U3, Nikon, Japan).

### 2.2 Animals

Male Sprague–Dawley rats (200–220 g) purchased from Beijing Vital River Laboratory Animal Technology Co. Ltd. (Licence No. SCXK (Beijing) 2019–0010) were kept at a controlled normal atmospheric temperature (20°C~25°C) and 40%~60% relative humidity under a light and dark cycle. Rats were fed a diet of natural ingredients and water ad libitum. Experimental procedures were conducted as per the Guide for the Care and Use of Laboratory Animals published by the US National Institutes of Health (NIH Publication No. 86‐23, revised 1996). All protocols were approved by the ethical review of the China Academy of Chinese Medical Sciences (animal ethics registration number: ERCCACMS21‐2111‐08, 08, November, 2021).

### 2.3 Preparation of DQJD Decoction

DQJD was conducted based on the literature [13]. Briefly, weigh a certain amount of decoction pieces (Table 1) and soak them in water for 30 min. After boiling with high heat, continue to decoct for 20 min to obtain a water decoction. Add water to the medicinal residues and continue to decoct for 20 min. Combine 2 times of water decoction and concentrate to 1 mg/mL crude drug, refrigerated for later use. Quality control of DQJD can be seen in the Appendix S1 (Figure S1).

### 2.4 Establishment of a CCH Model and Treatment

**FIGURE 1:**
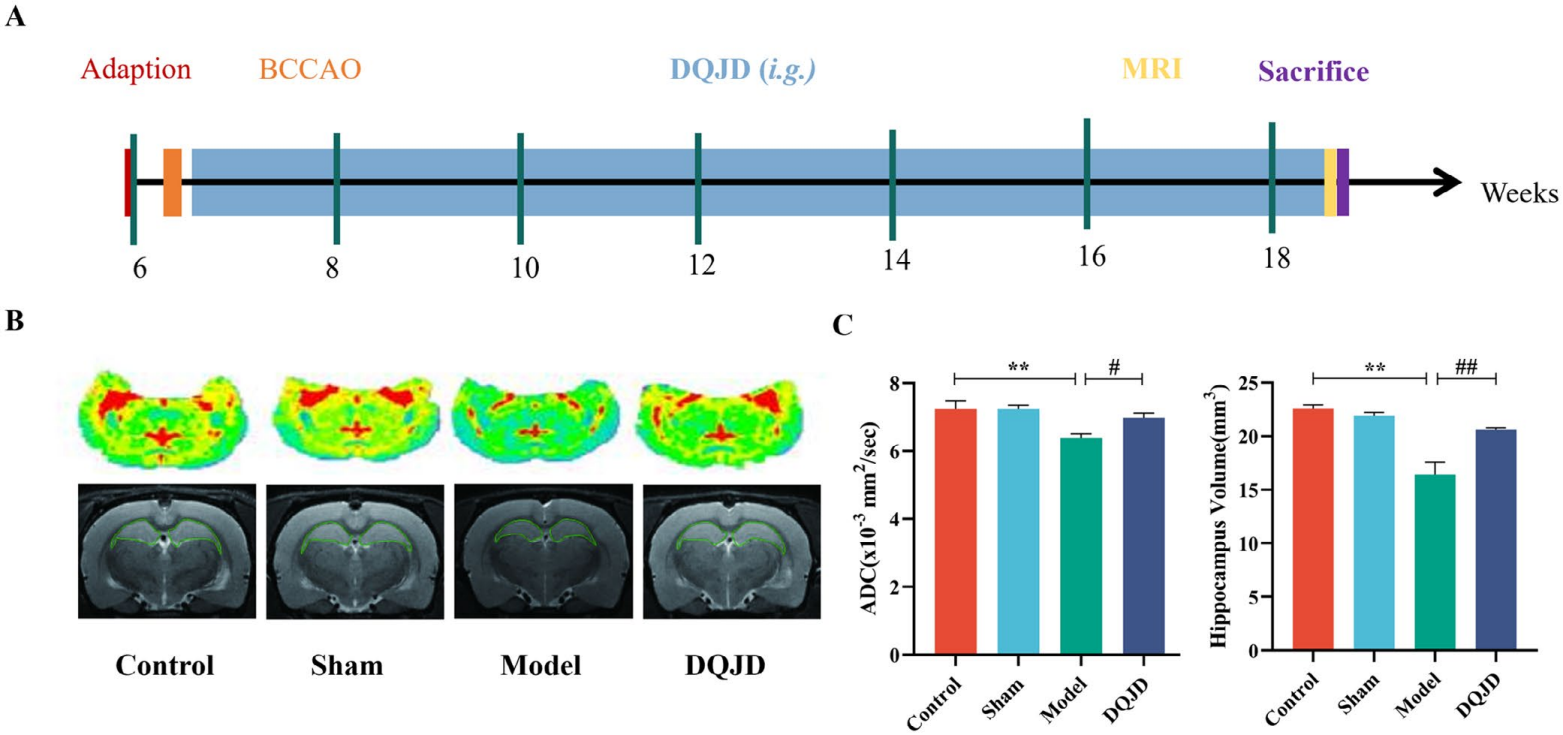
CCH model was established in rats and CBF damage in the brain was confirmed by MRI. (A) Schematic diagram of the experimental procedures. DQJD was administered via standardised intragastric gavage (i.g.). (B) Representative contrast and photographic images of brain perfusion. CBF maps and hippocampal volume scans of rats, respectively. (C) Quantification of apparent diffusion coefficient (ADC) and hippocampal volume (mean ± SD, *n* = 3). **p < 0.01 (versus control); ^#^ *p* < 0.05 (versus model); ^##^ *p* < 0.01 (versus model).

Animals were used for the study after 2 days of acclimatisation. Briefly, rats were separated into three groups at random: a control group (Control, *N* = 8), a sham group (Sham, *N* = 8), and a BCCAO operation group (BCCAO, *N* = 16). All rats in the BCCAO group were made into a CCH model by permanent ligation of bilateral common carotid arteries. Rats were anaesthetised by intraperitoneal injection of pentobarbital sodium solution at a concentration of 1% based on a dose of 45 mg/kg before surgery. According to previous studies [14], the right and left common carotid arteries were ligated 10 min apart, resulting in permanent ligation of the common carotid artery. The sham‐operated rat received the same surgical procedure apart from carotid occlusion. Maintain rat body temperature at 37°C ± 0.5°C using a thermostatic system until awake. No animals died during the operation, and 16 successfully modelled rats were allocated to the model group (Model, *n* = 8) and the DQJD group (DQJD, *n* = 8) utilising the random number table method. The technology roadmap of this study is shown in Figure 1A.

The rats in the DQJD group were administered the Chinese medicine decoction of DQJD via intragastric gavage at a dose of 6.34 g/kg, which was converted based on the amount commonly used in human clinical practice and the ratio of body surface area [15]. In the control, sham, and model groups, the rats were gavaged with the same volume of distilled water as the DQJD group once a day for 12 weeks. After a 12‐week DQJD intervention, neurobehavioural assessments and MRI were performed to evaluate cognitive function and hippocampal volumetric changes.

### 2.5 MRI Imaging

After 12 weeks of DQJD administration, MRI was performed to assess the hippocampal volume of rats. As previously described, we maintainesd anaesthesia at an isoflurane concentration of about 1.5%–2%, and adjusted the amount of anaesthesia according to the respiratory rate [16]. MRI was conducted at 7.0 T using a PharmaScan 70/16 system (BioSpec, Bruker, USA) equipped with a body coil for RF excitation and an inductively coupled surface coil placed on the skull for signal reception [17]. Spin‐echo sequences were acquired using the Paravision 6.0 operational processing system to record diffusion‐weighted imaging (DWI) with a repetition time of 3000 ms and an echo time of 39 ms. Four coronal slices of 0.65 mm thickness were obtained with a 35 × 35 mm field of view and a matrix size of 128 × 128. Diffusion weighting was performed using B augments of 0, 100, 200, 400, 800, 1200 and 1500 s/mm^2^ to allow an analysis of ADC. RadiAnt DICOM Viewer v2020.2.3 software was used to measure the hippocampal area of each layer and calculate the hippocampal volume based on the scanned layer thickness.

### 2.6 Histopathological Evaluation

Cortical and hippocampal tissues collected from three rats were fixed in 4% paraformaldehyde for ≥ 24 h and dehydrated using a graded ethanol series in an automated tissue processor, followed by paraffin embedding and sectioning at 5 μm thickness. Haematoxylin and eosin (HE) staining and mounting were carried out according to the standard protocol. Briefly, sections were stained with haematoxylin for 3 min, differentiated with 1% hydrochloric acid aqueous solution, and rinsed in running tap water. Subsequently, the sections were stained with eosin for 5 min. Then, the sections were dehydrated and cleared by absolute ethanol and xylene, respectively.

Standard histological assessments were performed by a pathologist unaware of the experimental conditions. Inflammation and tissue damage were confirmed using a light microscope (DM4000B, Leica, USA). The number of neurons was analysed by ImageJ software. All evaluations were done by a researcher blind to the experiment.

### 2.7 Transmission Electron Microscopy

Cerebral cortex specimens (1 mm^3^) were immediately immersed in pre‐chilled 2.5% glutaraldehyde (Lot.SBJ‐0639, Nanjing SenBeiJia Biological Technology Co. Ltd.) for primary fixation, followed by secondary fixation with 1% osmium tetroxide (Lot.18456, Ted Pella Inc). The fixed specimens were subsequently dehydrated by a succession of acetone (Lot.10000418, Sinopharm Chemical Reagent Co. Ltd.), gradually increasing in concentration until reaching 100% and embedded in epoxy resin (Lot.02660, SPI). Ultrathin sections (60 nm) were sliced and treated with uranyl acetate and lead citrate. Ultrastructural observation was performed by transmission electron microscopy (Lot.H7650, Hitachi Ltd.).

### 2.8 Determination of Serum TNF‐α, ICAM‐1 and COX‐2

After deep anaesthesia, fresh blood collected from the rat abdominal aorta was placed at 4°C for 5 h and then centrifuged at 4600 × g (4°C, 10 min), and the supernatant was collected and stored in a −80°C refrigerator. Rat ELISA kits of TNF‐α (Lot.EK382/3), COX‐2 (Lot.CSB‐E13399r), and ICAM‐1 (Lot.PI495) were purchased from Multi Sciences (Lianke) Biotech Co. Ltd. (Zhejiang, China), CUSABIO Co. Ltd. (Wuhan, China), and Beyotime Biotech Inc. (Shanghai, China), respectively. The concentrations of TNF‐α, ICAM‐1, and COX‐2 were detected with ELISA kits, determined according to the commercial instructions of the kit.

### 2.9 Transcriptomics Analysis

Total RNA of cortex tissues was extracted using the Trizol reagent according to the manufacturer's protocol. RNA purity and concentration were quantified using the NanoDrop 2000 spectrophotometer (Thermo Scientific, USA). RNA integrity was assessed through the Agilent 2100 Bioanalyser (Agilent Technologies, Santa Clara, CA, USA).

The library construction, sequencing, and data analysis were accomplished by Shanghai Ouyi Biomedical Technology Co. Ltd. The raw reads produced in high‐throughput sequencing are fastq format sequences. To acquire high‐quality reads that can be used for the following analysis, quality filtering of the raw reads is indispensable. Firstly, the Trimmomatic [18] software was employed for quality control and adapter sequence removal. Through this process, low‐quality bases and ambiguous N bases were filtered out, ultimately retaining high‐quality clean reads for downstream analysis. The clean reads were compared to the reference genome of the species using hisat2 [19] with default software parameters, and the samples were assessed by the genome comparison rate. Clean reads were compared to the reference genome and stored as binary files, that is, bam files. Gene FPKM [20] expression values were quantified using the cufflinks software [21]. In calculating the expression differences of genes, the number of reads that fell to each gene in each sample was obtained using the htseq‐count [22] software. The data were normalised using the estimateSizeFactors function of the DESeq (2012) R package [23], and the nbinomTest function was used to calculate the difference comparison of *p*‐value and foldchange values. Differentially expressed genes (DEGs) with *p*‐values ≤ 0.05 and foldchange ≥ 1.5 were selected. The DEGs were also subjected to unsupervised hierarchical clustering to demonstrate the expression pattern of DEGs among various samples using a heat map format.

### 2.10 Identification of Potential Targets for DQJD Against CSVD and PPI Network Construction

We utilised the Traditional Chinese Medicine Systems Pharmacology database [24] to identify active ingredients in DQJD with OB ≥ 30% and DL ≥ 0.18 as the evaluation criteria. The potential targets of DQJD were identified using the SwissTargetPrediction database [25]. The CSVD‐related targets were obtained from GeneCards [26], DisGeNET [27], and Online Mendelian Inheritance in Man (OMIM) [28]. The intersection targets of DQJD and CSVD were obtained, and Venn diagrams were drawn using the bioinformatics platform [29].

The overlapping target genes were imported to the STRING database [30] to create a protein–protein interaction (PPI) network. The species was set to ‘*Homo sapiens*’, the protein interaction score was set as ‘medium confidence’ (> 0.4), and the remaining parameters were left as default. Cytoscape 3.10.1 software was used for the construction and topological analysis of the network of common targets [31]. Essential topological parameters, including degree centrality, closeness centrality and betweenness centrality, were selected to screen the core targets on the basis of the PPI network. The compound‐target network was constructed using Cytoscape 3.10.1 software to investigate the complicated interaction between the ingredients and the core targets. The main active ingredients were identified based on the topological parameters.

### 2.11 GO and KEGG Pathway Analysis

The Gene ontology (GO) [32, 33] and Kyoto Encyclopedia of Genes and Genomes (KEGG) [34] enrichment analyses were conducted to investigate the roles of candidate targets for DQJD against CSVD and identify the biological functions or pathways primarily associated with DEGs between the DQJD‐treated and CCH model rats.

### 2.12 Molecular Docking

To confirm the result of transcriptomics, molecular docking was carried out between the Rxrg and mandenol, a component of DQJD, by AutoDockTools (version 1.5.7) and AutoDockVina (version 1.2.0). The protein structures of the Rxrg were retrieved from the PDB database (http://www.rcsb.org/), and the SDF file of mandenol was obtained from PubChem (https://pubchem.ncbi.nlm.nih.gov/). The optimal docking poses were visualised and analysed using PyMOL 2.0 software and Discovery Studio Visualizer 2021.

### 2.13 RT‐qPCR Analysis

Total RNA was extracted from each group of rat focal tissue by the TRIzol method. The internal reference protein was GAPDH, and the reaction system and parameters were set up according to the Promega kit (Promega Corporation, Madison, USA, Lot:0000480098). The Ct values of the target gene and GAPDH were obtained by the sample point fitting method, the relative expression was calculated by the 2^−ΔΔCt^ method, and the primers were synthesised by Bioengineering (Shanghai) Co. Ltd. The sequences of the primers are listed in Table 2.

**TABLE 2:** Primer sequence.

Gene	Primer	Sequences
*GAPDH*	F	CTGGAGAAACCTGCCAAGTATG
	R	GGTGGAAGAATGGGAGTTGCT
*SLC27A2*	F	AGGTGAGGTTGGACTCTTGATTTGC
	R	GGAGATCGCCACTGTTGAAGTAGAC
*RXRG*	F	CGAATCCTACGGCGACATGAGTG
	R	AACAAGGGTGAAGAGCTGCTTATCC

### 2.14 Western Blot Analysis

Cortical tissues (100 mg) from spare rats were homogenised in RIPA lysis buffer (Solarbio, R0010) containing 1 mM PMSF protease inhibitor (Solarbio, R0100), followed by 10 min lysis on ice. The homogenate was centrifuged at 12,000 × g (radius: 86 mm) for 10 min at 4°C. The supernatant was collected and stored at −80°C. BCA protein quantification kit (Solarbio, Beijing, China) was used to detect the total protein concentration; the separation gel and concentrated gel were prepared; SDS‐PAGE electrophoresis (Solarbio, Beijing, China) was carried out; and the protein was transfer on PVDF membrane. The blocking incubation was carried out using the one‐step western kit HRP (Cowin Biotech, Beijing, China). The membrane was incubated with blocking buffer for 5 min at room temperature and washed by adding the configured antibodies, VEGF‐α (1:1000, Proteintech), RXR‐γ (1:800, Proteintech) and PPAR‐γ (1:5000, Proteintech), incubated for 90 min at room temperature and washed for 10 min. ECL chemiluminescence (Solarbio, Beijing, China) was used to develop the bands.

### 2.15 Statistical Analysis

Statistical data were analysed using GraphPad Prism version 8 software. Experimental data were expressed as mean ± standard deviation (SD). Shapiro–Wilk test was performed to assess the normality of the data distribution, and *p*‐value ≥ 0.05 suggests that the data closely follow a normal distribution. A two‐tailed Student's *t*‐test was employed to detect statistical differences between two groups, and one‐way analysis of variance (ANOVA) followed by Tukey's post hoc test was utilised to analyse the statistically significant differences among multiple groups. Statistical significance was defined as *p* < 0.05.

## 3 Results

### 3.1 Effect of DQJD on MRI and Brain Histomorphology in CCH‐Induced Rats

Behavioural tests, including the Open Field Test and Morris Water Maze Test, were conducted to evaluate the effects of DQJD on neurological recovery after CCH. As shown in Figures S2 and S3, compared to control and sham groups, the model group exhibited prolonged dwell time in the peripheral zone of the OFT, increased total movement and reduced activity frequency without reaching statistical significance. On the contrary, rats in the DQJD group significantly reduced the moving time on the four edges and increased the number of activities in the Open Field Test, as well as an increased dwell time in the target quadrant in the Morris Water Maze Test. It demonstrated that DQJD treatment ameliorated the impairment of learning and memory induced by CCH. DWI, an imaging method, reflects the irregular motion of water molecules within the tissue and changes in the diffusion capacity of water molecules will result in changes in signal intensity [35]. The apparent diffusion coefficient (ADC) can be used to quantify the diffusion capacity of water molecules. Decreased ADC indicates the alterations in the ratio of intracellular and extracellular volume as ischaemia‐stimulated energy deficiency and membrane pump failure allow water to permeate from the extracellular to the intracellular space, leading to cytotoxic oedema [35]. The diffusion coefficient and hippocampus volume of the brains in the model rats were decreased relative to the control and sham groups (Figure 1B,C), which indicated the successful establishment of the CCH model. No significant oedema was seen on the MRI in the DQJD group; the hippocampal volume was smaller than that of the sham rats, and the border definition was slightly inferior to that of the sham rats (Figure 1B). Our results obtained from MRI showed that in DQJD rats, the ADC and hippocampus volumes were elevated by 9.4% (*p* < 0.05) and 25.6% (*p* < 0.01), respectively, versus the model rats (Figure 1B). An ischaemic core and a peripheral area are defined as the ischaemic penumbra with reduced CBF while preserving energy metabolism [36]. According to the definition, the ischaemic penumbra is a severely under‐perfused but salvageable brain region around the ischaemic core and an important target for current neuroprotective interventions [37]. Overall, cerebral samples observed in the ischaemic penumbra region of CCH rats and corresponding regions of control, sham and DQJD rats (mainly focused on cortical and hippocampal tissues) were collected for further experiments.

### 3.2 DQJD Ameliorates the Inflammatory Phenotype and Neuronal Death in CCH‐Induced Rats

**FIGURE 2:**
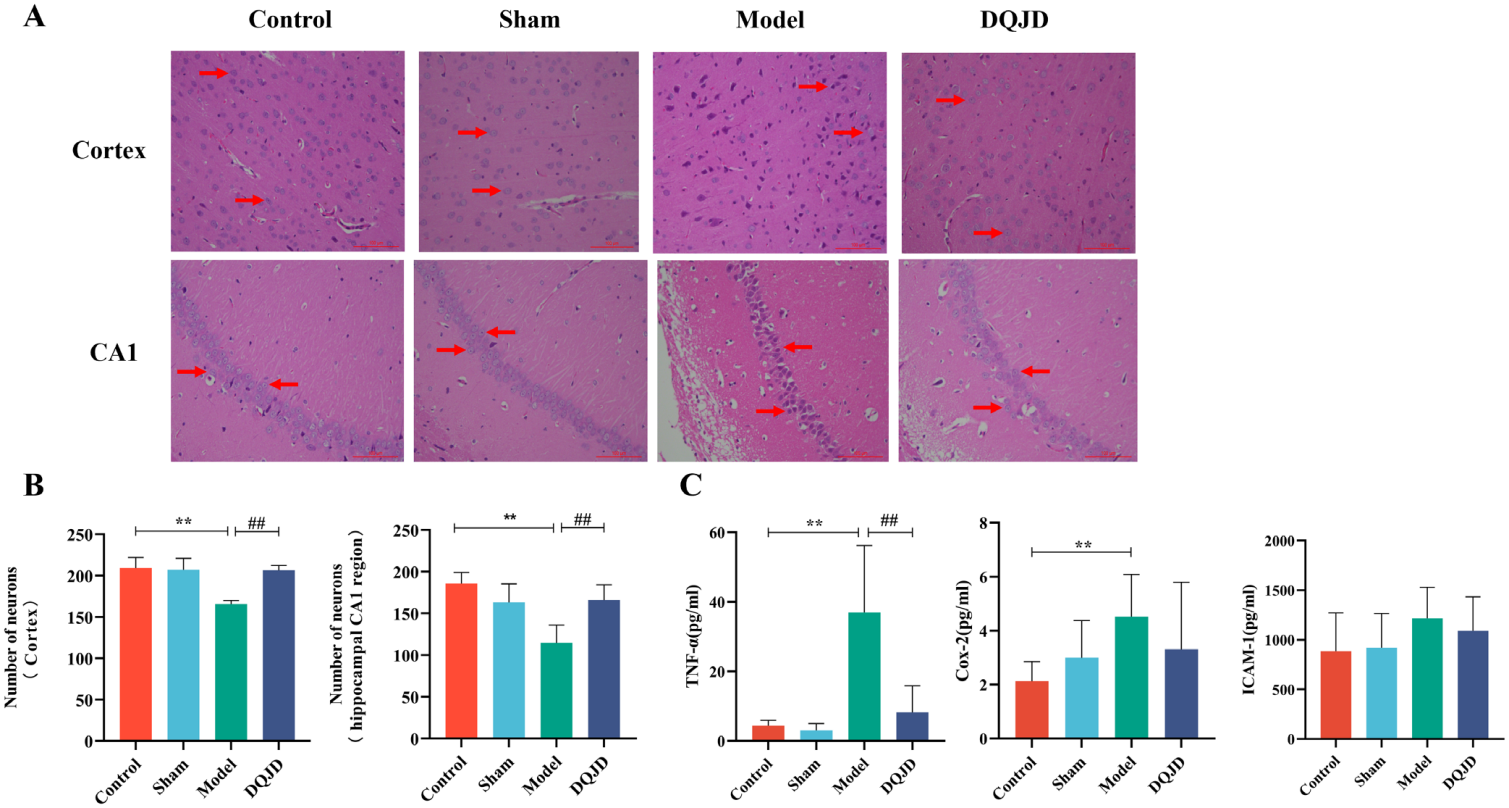
DQJD reduces CCH‐induced neuronal death and inflammation levels in vivo. (A) HE staining of cortex (scale bar = 100 μm) and hippocampal CA1 (scale bar = 100 μm). (B) The number of neurons in the cortex and hippocampal CA1 was evaluated by Image J software (*n* = 3). (C) Serum levels of TNF‐α, COX2, and ICAM‐1 were measured by ELISA (mean ± SD, *n* = 8). **p < 0.01 (versus control); ^##^ *p* < 0.01 (versus model).

Consistent with the aforementioned MRI findings, previous studies have reported that CCH‐induced pathological damage demonstrated greater severity in the hippocampal CA1 and cortex [38]. Thus, HE staining of the hippocampal (CA1) and cortex was conducted to assess the neuroprotective effects of DQJD. The HE staining results showed that neurons in the cerebral cortex were structurally intact, and the nuclei of neurons were round in control and sham rats (Figure 2A). As shown in Figure 2A, compared with the control and sham groups, fuzzy cell structure, unobvious nucleoli, shrinkage of the cell body, vacuolar degeneration, and nuclear atrophy or even necrosis were observed in model rats. In contrast with the model group, DQJD treatment improved the morphology of cerebral cortex neurons, which showed intact cellular structures and clear cell membranes, as presented in Figure 2A. The neurons in the hippocampal CA1 region of control and sham group rats were closely connected with intact cytoarchitecture. Compared with the control and sham groups, the nerve cells in the CA1 area of the hippocampus of the model group were loosely arranged with incomplete cell structure, and the cell numbers were markedly reduced. In contrast to the model group, DQJD treatment improved the arrangement of cells in the hippocampal CA1 region, with relatively complete cell structure (Figure 2A). The neuronal counts were quantified alongside the observation of cellular morphology to evaluate the neuroprotective effects of DQJD. Compared with the control group, the number of nerve cells in the cortex and hippocampal CA1 region significantly decreased in model rats (*p* < 0.01). In contrast with the model group, DQJD treatment increased the number of nerve cells in the cortex and hippocampal CA1 region (*p* < 0.01) (Figure 2B). These results suggest that BCCAO causes vascular neural unit damage and brain tissue ischaemia and hypoxia through localised reduction of cerebral blood flow, which ultimately triggers neuronal death. DQJD administration increased neuronal density in the cortex and hippocampus of CCH‐induced rats.

Neuroinflammation exerts a major responsibility in the pathogenesis of CCH [39]. To assess the anti‐neuroinflammatory effects of DQJD, the levels of pro‐inflammatory factors (TNF‐α, COX‐2 and ICAM‐1) were examined. The results suggested that the concentrations of TNF‐α and COX‐2 in the model group were particularly higher than those in the control rats (*p* < 0.01). Although ICAM‐1 expression showed an upward trend compared to control rats, no statistically significant differences were observed (*p* > 0.05, Figure 2C). In contrast, DQJD administration only induced a statistically significant reduction in the TNF‐α level (*p* < 0.01). These findings suggest that DQJD may accelerate cortical and hippocampal repair in CCH models by suppressing pro‐inflammatory mediators.

### 3.3 Effect of DQJD on the Ultrastructure of the Cortex in CSVD Rat

**FIGURE 3:**
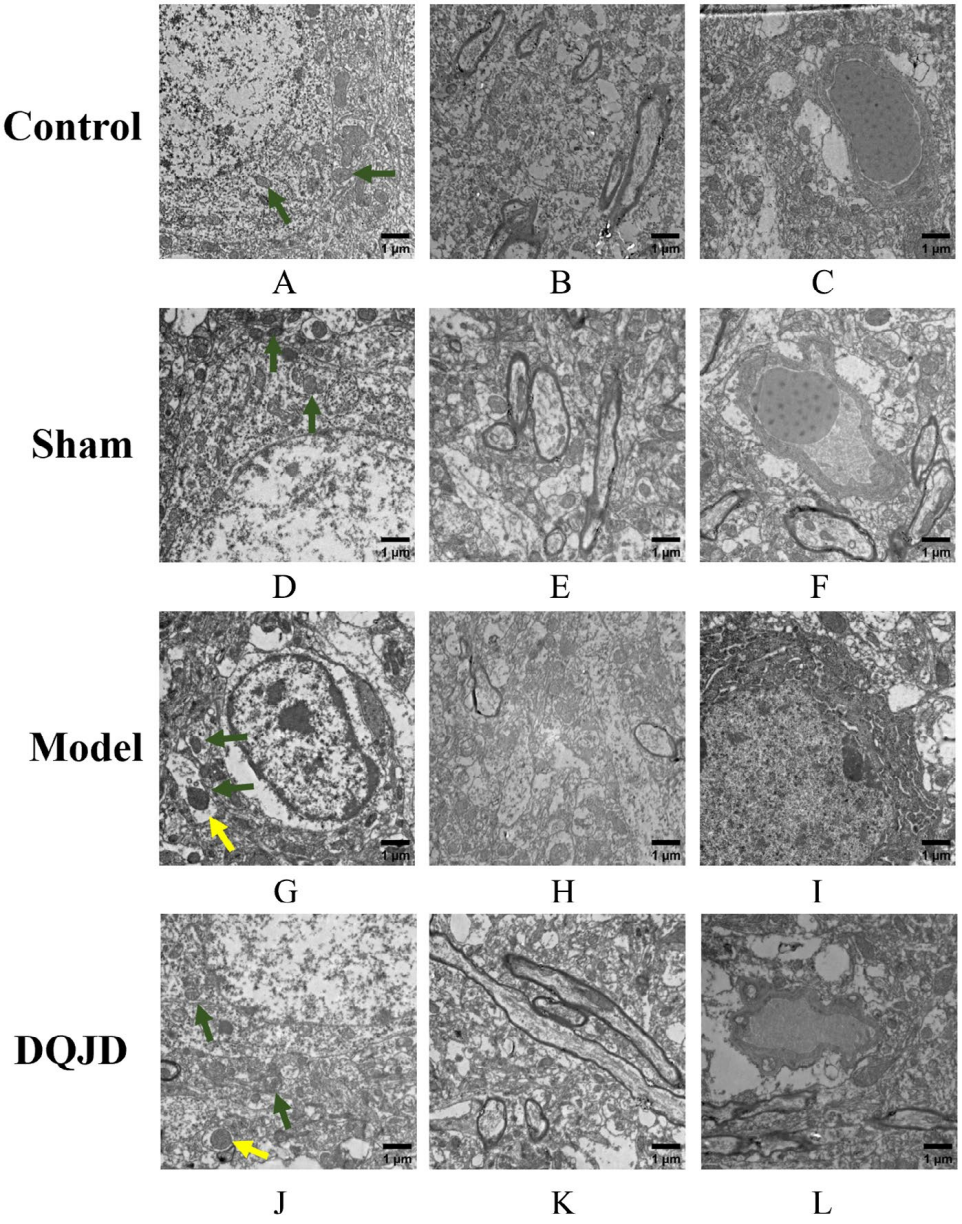
Ultrastructure of cortex in each group of rats under transmission electron microscope. (A–C) blank group rat cortex region. the structures of organelles and mitochondria are complete. (D–F) sham group rat cortex region. (G–I) model group rat cortex region, the structures of organelles and mitochondria are damaged. (J–L) DQJD group rat cortex region, DQJD improved the structure of organelles and mitochondria. In TEM, green arrows represent mitochondria and yellow arrows represent autophagosome. *n* = 3.

Following HE staining analysis, brain tissues from each experimental group were examined via transmission electron microscopy to assess the ultrastructural neuroprotective effects of DQJD on neuronal architecture. In the control and sham‐operated groups, neurons exhibited intact nuclear architecture with large, rounded nuclei with homogeneous chromatin distribution. Cytoplasmic organelles, including the Golgi apparatus, rough endoplasmic reticulum and mitochondria, were distinctly observed. Mitochondria displayed circular/ovoid morphology with intact cristae architecture, while lipofuscin deposits were rarely detected. Myelin sheaths maintained compact organisation, and vascular basement membranes demonstrated tight apposition to extracellular matrix (ECM) components (Figure 3A–F). The cellular contours in the model group exhibited blurring, with irregularity in nuclear morphology. Mitochondrial cristae displayed fractures and disintegration, with partial cristae disappearance resulting in vacuolated or hyaline structures. Lipofuscin and autophagosome accumulation appeared. The vascular basement membrane exhibited widening of the extracellular matrix interstitial space, accompanied by myelin sheath reduction and loosened alignment (Figure 3G–I). Compared with the model group, DQJD exhibited reduced lipofuscin and autophagosome accumulation, diminished mitochondrial vacuolation, and a non‐significant widening of the vascular basement membrane and extracellular matrix interstitium (Figure 3J–L). These findings indicate that CCH induces brain tissue damage, neurodegeneration and mitochondrial impairment. Conversely, DQJD treatment preserved mitochondrial ultrastructure while mitigating cerebral cortical ischaemia–reperfusion injury in rats.

### 3.4 RNA‐Sequencing Highlights Rat Cortex Damage in CCH‐Induced Models

**FIGURE 4:**
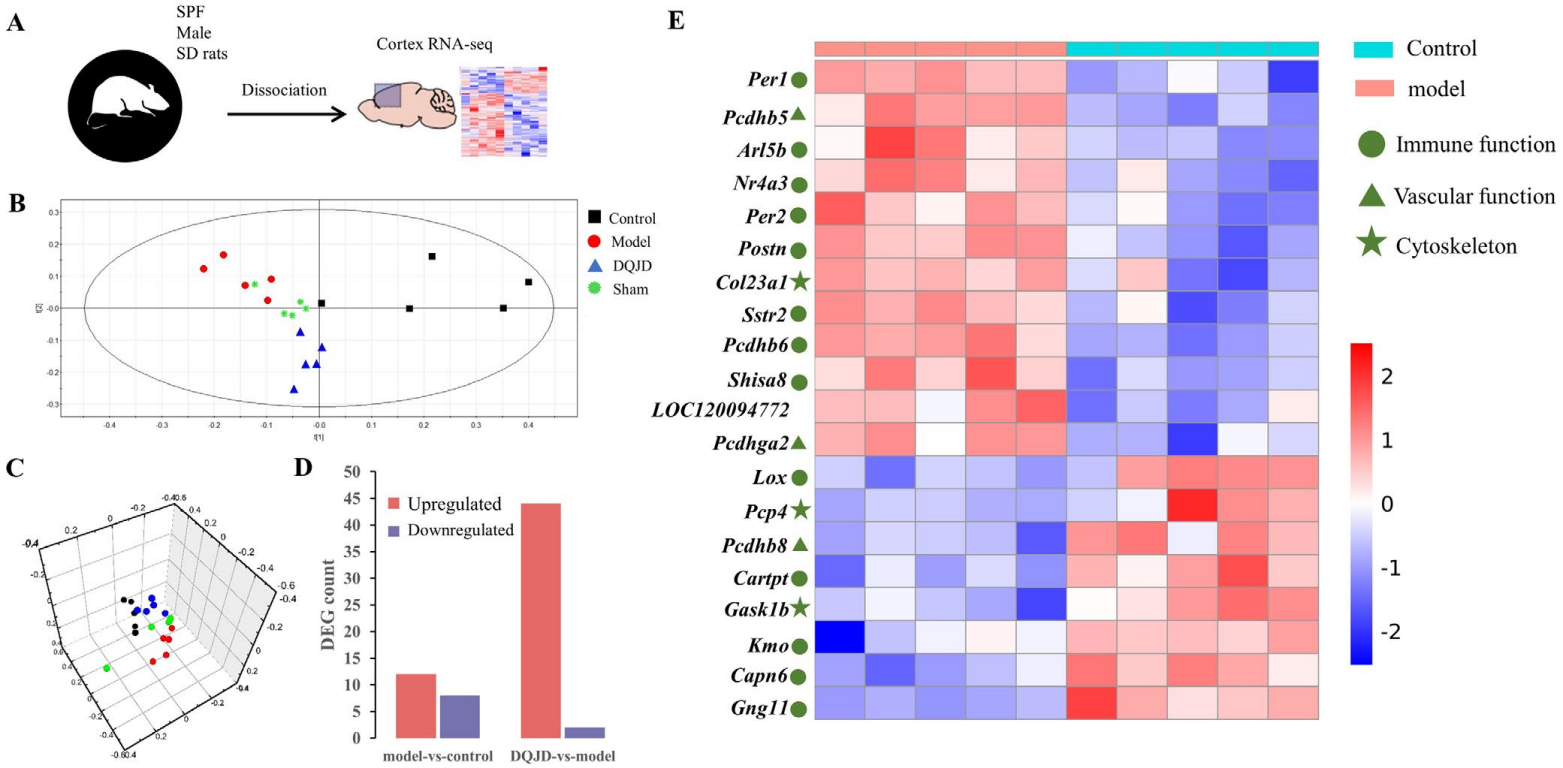
DQJD treatment orchestrates cerebral cortex transcriptome in CCH‐induced rats. (A) Schematic diagram of the experimental setting: RNA‐seq of cortex from SPF male Sprague–Dawley rats (Control, Sham, Model and DQJD). (B) PCA analysis of each group (*n* = 5). (C) 3D PCA analysis of each group (*n* = 5). (D) Bar graph shows the number of DEGs in each group (up‐regulated in red, down‐regulated in blue, *n* = 5). (E) Heat map shows the gene differences between the model and control groups (*n* = 5, *p* ≤ 0.05, FC > 1.5). The list of genes with symbols (left) indicates their functional annotation (top right). Each column is a sample and each row is a gene. *Z*‐scores were calculated based on normalised gene counts (up‐regulated in red, down‐regulated in blue).

**FIGURE 5:**
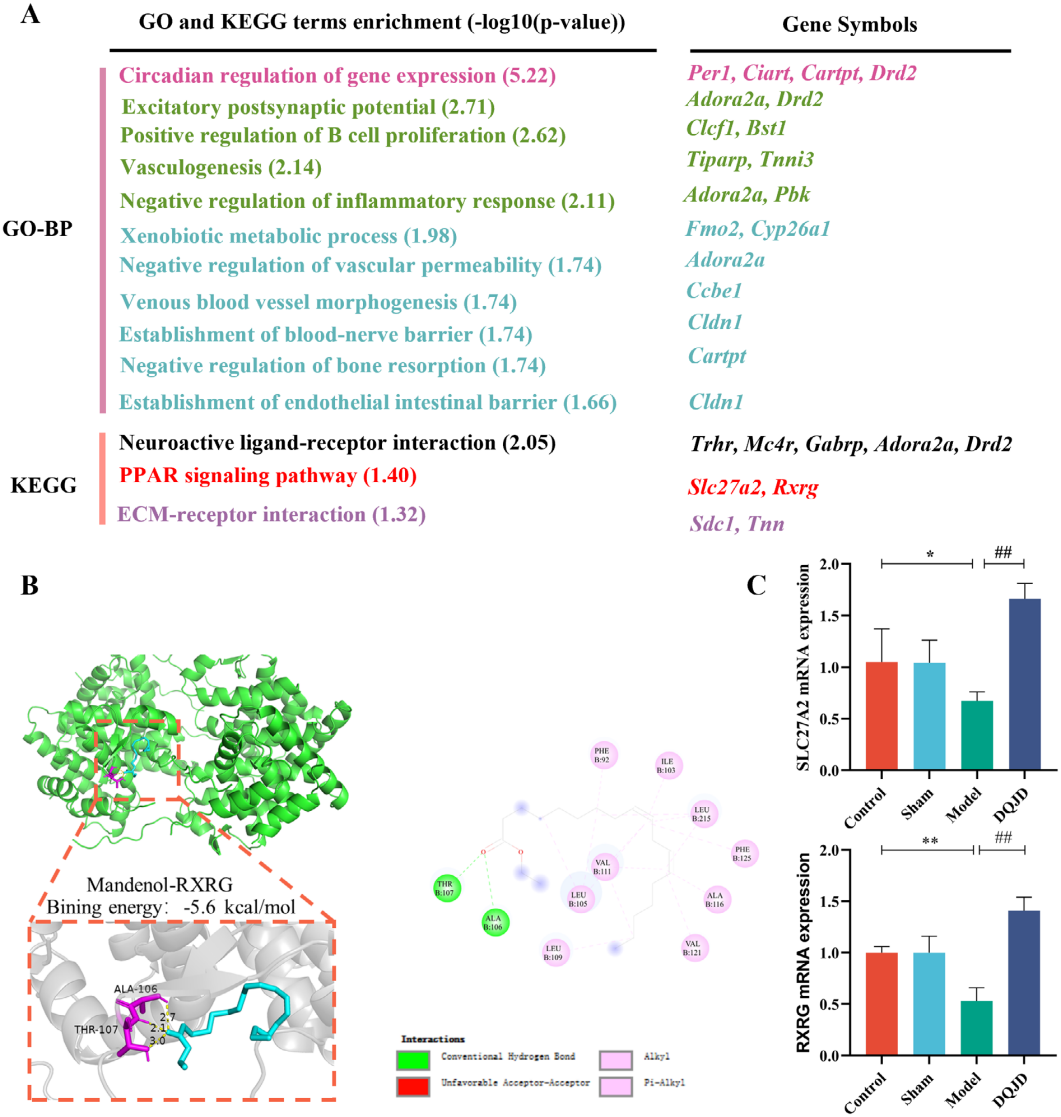
List of GO and KEGG enrichment analyses in DQJD‐treated rats compared to CCH‐induced rats sorted after their fold changes. (A) Compared to the model, significant gene ontology (GO) terms and KEGG pathways were enriched in DQJD with the respective −log_10_ (*p*‐value). Enriched genes are shown on the right (*n* = 5). (B) The interactions between mandenol and RXRG. (C) Confirmation of RNA‐seq results by RT‐qPCR (mean ± SD, *n* = 5). *p < 0.05 (versus control), **p < 0.01 (versus control); ^##^ *p* < 0.01 (versus model).

To further investigate the molecular mechanisms involved in the pathological alterations of DQJD in CCH rats, we performed RNA‐seq analysis on the cortical tissue (Figure 4A). Principal component analysis (PCA) highlighted the different gene expression profiles of cortical tissues in the four groups of rats (Figure 4B,C). Combining the two screening conditions of *p* ≤ 0.05 and fold change ≥ 1.5, we identified the DEGs between groups (Figure 4D). Interestingly, 20 DEGs (12 up‐regulated and 8 down‐regulated) were screened in the model versus the control group. DQJD treatment resulted in 46 DEGs (44 up‐regulated and 2 down‐regulated) versus the model group. Notably, unique gene expression patterns emerged in the cortex of the model group versus the control rats, including those related to immune function (e.g., *Per1, Arl5b, Nr4a3, Per2, Postn, Sstr2, Pcdhb6, Shisa8, Lox, Carpt, Kmo, Capn6* and *Gng11*), vascular function (*Pcdhb5, Pcdhga2 and Pcdhb8*) and cytoskeleton (*Col23a1, Pcp4* and *Gask1b*) related genes (Figure 4E). Next, we used GO and KEGG enrichment analysis to characterise the functional changes in gene expression in rat cortical tissue following DQJD treatment in terms of inflammation and vascularity. In comparing the model and DQJD, we first performed GO‐BP analysis (Figure 5A), and some GO terms related to inflammation and vascular regulation were positive regulation of B cell proliferation (*Adora2a*, *Drd20*), vasculogenesis (*Tiparp*, *Tnni3*), negative regulation of inflammatory response (*Adora2a*, *Pbk*), negative regulation of vascular permeability (*Adora2a*), venous blood vessel morphogenesis (*Ccbe1*), and establishment of blood‐nerve barrier (*Cldn1*). Second, according to KEGG enrichment analysis, neuroactive ligand‐receptor interaction (*Trhr, Mc4r, Gabrp, Adora2a* and *Drd2*), PPAR signalling pathway (*Slc27a2*, *Rxrg*), and ECM‐receptor interaction (*Sdc1*, *Tnn*) were significantly different between the two groups. To ascertain the RNA‐seq results, we selected 2 DEGs that were enriched in the PPAR signalling pathway. According to SwissTargetPrediction results of compounds screened by TCMSP, Rxrg is the target of mandenol. Thus, molecular docking was conducted to evaluate the binding affinity between mandenol and Rxrg (Figure 5B). The docking result indicated that mandenol can bind well with Rxrg (PDB ID:2GL8; Bing energy: −5.6 kcal/mol). The up (down) regulation results of these genes measured by RT‐qPCR were the same as RNA‐seq (Figure 5C). In conclusion, the functional analysis of DEGs suggested that DQJD may ameliorate CCH‐induced pathological injury through the PPAR pathway.

### 3.5 Network Pharmacology Analysis

**FIGURE 6:**
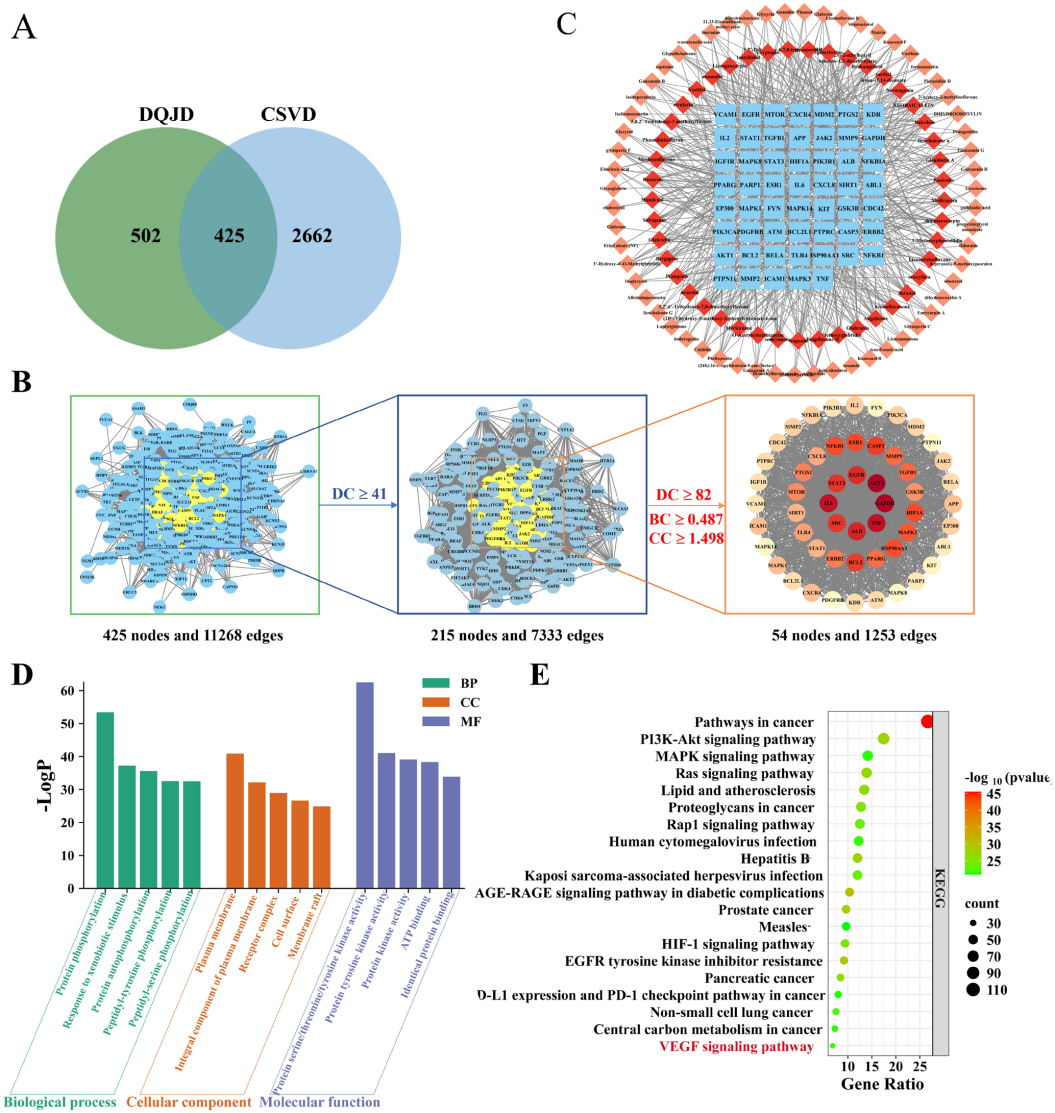
The molecular mechanism of the anti‐CSVD activity of DQJD as predicted based on the network pharmacology approach. (A) The Venn diagram shows common candidates between CSVD and DQJD identified targets. (B) The process of topological screening for the PPI. (C) “The compounds‐core targets” network. (D) The top 5 BP terms, CC terms, and MF terms of GO enrichment analysis are shown as green, orange, and purple bars, respectively. (E) Top 20 enriched KEGG pathways of common candidate targets.

To investigate the molecular mechanisms underlying DQJD's therapeutic effects on CSVD, network pharmacology analysis was conducted alongside transcriptomic profiling to identify potential pathways involved in this intervention. On the basis of the TCMSP database, 109 active compounds of DQJD were obtained. We obtained 927 potential gene targets of the active ingredients of DQJD from the SwissTargetPrediction database and 3087 CSVD‐related targets from GeneCards, OMIM, and DisGeNET databases. A total of 425 intersection targets were obtained using the bioinformatics platform, which were considered candidate target proteins for DQJD in the treatment of CSVD (Figure 6A). As shown in Figure 6B, a core PPI network with 54 core targets was subsequently identified according to topological analysis with ≥ 2 times the median degree centrality as the screening threshold.

Topological analysis revealed that the top targets with the highest degree values were AKT1, IL6, TNF and ALB, which were considered to be the hub targets. In the topological analysis of the compound‐target network, the top 10 compounds with the highest degree values were baicalein, glyasperin B, (2R)‐7‐hydroxy‐5‐methoxy‐2‐phenylchroman‐4‐one, angelicone, acacetin, epiberberine, Norwogonin, Moslosooflavone, Cryptopin and Isotrifoliol. These compounds may be the main ingredients in DQJD that exert anti‐CSVD effects (Figure 6C).

To explore the underlying mechanisms of DQJD intervention in CSVD, GO and KEGG enrichment analyses were performed. GO enrichment analysis suggested that intersection target genes were mainly enriched in protein phosphorylation, plasma membrane, and protein serine/threonine/tyrosine kinase activity (Figure 6D). The KEGG analysis of the intersection genes demonstrated that multiple pathways were involved in the anti‐CSVD effects of DQJD, including the PI3K‐Akt signalling pathway, MAPK signalling pathway, Ras signalling pathway, Rap1 signalling pathway, HIF‐1 signallingsignaling pathway, and VEGF signaling pathway (Figure 6E). A vast amount of research has confirmed that the VEGF signalling pathway is implicated in the development and progression of CSVD. VEGF‐α, a growth factor with angiogenic activity [40], promotes vascular endothelial cell development and proliferation and has been shown to enhance synaptic plasticity, which is beneficial in experimental stroke. The upregulation of VEGF‐α reduces neuronal damage in ischaemic rats and improves neurobehavioural functions [41]. Collectively, these data indicate that the neuroprotective mechanism of DQJD in CSVD treatment potentially involves VEGF pathway activation.

### 3.6 DQJD Upregulates the Expression of Proteins Related to RXR‐γ/PPAR‐γ/VEGF‐α Pathway

**FIGURE 7:**
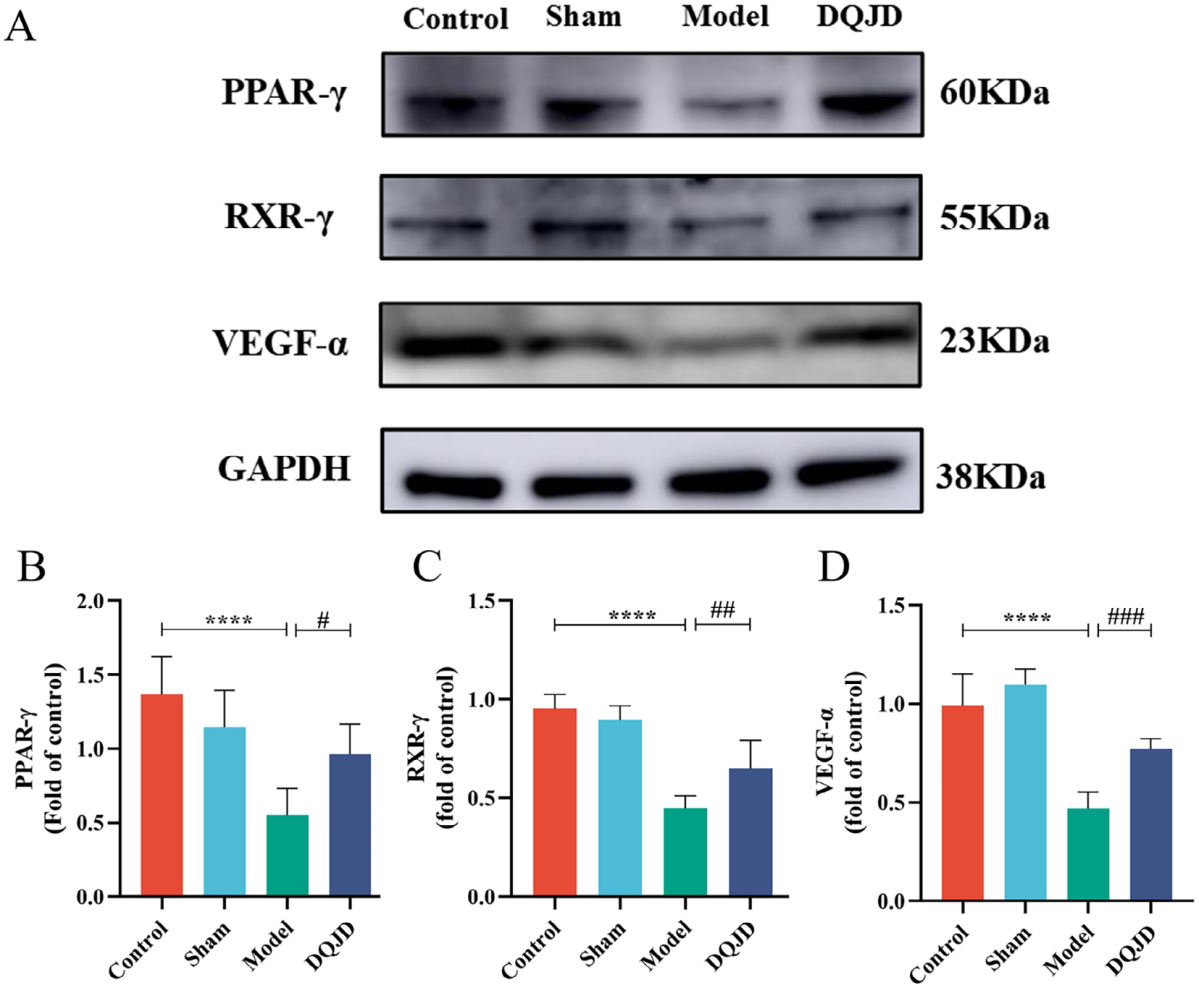
Effects of DQJD‐mediated RXR‐γ/PPAR‐γ/VEGF‐α activation in CCH‐induced rats. (A) Representative immunoblots of cortical RXR‐γ, VEGF‐α and PPAR‐γ. GAPDH was used as a control reference protein. (B–D) Data were expressed as a multiple of the density change compared to the control tissue by Image J software (mean ± SD, *n* = 5), ****p < 0.0001 (versus control); ^#^ *p* < 0.05 (versus model); ^##^ *p* < 0.01 (versus model); ^###^ *p* < 0.001 (versus model). PPAR‐γ, Peroxisome proliferator‐activated receptor gamma; RXR‐γ, Retinoid × receptor gamma; VEGF‐α, Vascular endothelial growth factor α.

Integrating transcriptomic profiling and network pharmacology analysis, we postulate that DQJD may exert therapeutic effects against CSVD through modulation of the PPAR‐γ/VEGF‐α signalling pathway. To further explore whether DQJD regulated the PPAR‐γ/VEGF‐α signalling pathway, the expression of relative proteins in brains was detected. The transcriptional process can be activated only after RXR‐γ binds to PPAR‐γ, which in turn regulates the expression of VEGF‐α [42]. Therefore, we detected the level of RXR‐γ to figure out whether the activation of PPAR‐γ would trigger the process. The result confirmed that RXR‐γ was significantly downregulated in the model rats, while the expression of RXR‐γ in DQJD was similar to that in the sham rats (Figure 7A,B). Compared with the control group, the expression levels of PPAR‐γ and VEGF‐α were significantly decreased in CSVD rats, suggesting that the RXR‐γ/PPAR‐γ/VEGF‐α signalling pathway was inhibited in the rats after ligation of the common carotid (Figure 7A–D). On the contrary, PPAR‐γ and VEGF‐α expression were remarkably increased after the administration of DQJD. Taken together, DQJD could improve CSVD by activating the RXR‐γ/PPAR‐γ/VEGF‐α signalling pathway.

## 4 Discussion

CSVD, aging cerebral microangiopathy, are associated with gait disturbances and mood disorders [43]. Currently, one of the most critical pathophysiological mechanisms of CSVD is endothelial dysfunction and BBB leakage [44]. Endothelial dysfunction also causes vascular structural and functional disorders that allow leakage from the blood vessels. Further damage can cause impaired autoregulation, in which the vessels are unable to dilate to maintain perfusion, ultimately leading to cerebral ischaemia. Therefore, protection of endothelial function and enhancement of the BBB integrity are the key mechanisms underlying the treatment of CSVD, offering unique advantages. Current Western medical interventions for CSVD primarily involve early stage therapeutic strategies, including thrombolytic agents, antihypertensive drugs and antiplatelet therapy. However, these approaches demonstrate suboptimal therapeutic efficacy and are associated with significant adverse effects [5]. According to TCM, CSVD is assigned to the categories of stroke, dementia and vertigo. It was identified as a disease of essential empty and out solid; qi‐deficiency is based, blood stasis is a symptom; qi deficiency and blood stasis are important pathogenesis of CSVD [5]. DQJD, recorded in a traditional Chinese medicine classic of more than 800 years ago, is commonly applied for CSVD treatment clinically [45]. It can cultivate healthy qi and blood while maintaining unobstructed channels and collaterals. DQJD demonstrates a multi‐target regulatory capacity, notably suppressing apoptosis‐related proteins (e.g., Caspase3, P53) while modulating neuroinflammation and vascular integrity [6]. This holistic mechanism aligns with the systemic nature of CSVD pathology, differing from single‐target Western drugs. In this study, based on a CCH‐induced CSVD rat model, a combination of transcriptomics, network pharmacology and experimental validation was initially used to explore the active ingredients and molecular mechanism of DQJD in treating CSVD. This study confirms that DQJD contains a variety of anti‐CSVD active ingredients and preliminarily elucidates its mechanism, providing additional evidence for the promotion of DQJD in clinical applications and the development of new drugs for the treatment of CSVD.

Globally or focally decreased CBF may contribute to the development of vascular cognitive impairment due to persistent cerebral hypoperfusion. Considering that the enhancement of the angiogenic process underscores the function of DQJD in the CCH‐induced model, the finding of accelerating the induction of new capillary formation and recovery of neurological function sheds insight on a neurovascular method to therapy and strengthens the understanding of the relationships among immune, vascular, and nervous system in CSVD patients [46]. In vivo research demonstrated that CBF was significantly reduced in the cortex and hippocampus after CCH manipulation and gradually returned to normal levels within 12 weeks via DQJD intervention. The observation of the CBF assay was in line with previous results [47]. The mechanism for the persistent decline in CBF in the early stages of the CCH model may be reduced angiogenesis, decreased vascular maturation and stability, and disruption of the BBB. DQJD intervention can improve the CBF in the CCH model. The enhancement of the angiogenic process mediated by DQJD in the CCH model not only promotes neovascularisation but also accelerates neurological functional recovery, unveiling a novel neurovascular therapeutic strategy that synergises vascular remodelling with neural repair [46]. By MRI, we identified that CCH damage to the brain was concentrated in the hippocampus and the cortex. Collectively, the rat model of CCH was successfully established in the present study, and DQJD treatment improved the CBF in the CCH model.

The hippocampal CA1 region and cortical neuronal damage have been identified as the primary pathological alterations induced by CCH, contributing to cognitive impairment [48]. Histopathological analysis revealed exacerbated neuronal damage in various brain regions of the model group, while DQJD treatment improved neuronal alignment, significantly increased cell density and relatively preserved cellular architecture. Transmission electron microscope analysis further revealed that ultrastructural analysis of the model group demonstrated mitochondrial cristae disruption/fragmentation, autophagosome accumulation, and widened extracellular matrix spacing within the vascular basement membrane. However, compared to the model group, the DQJD‐treated group exhibited reduced lipofuscin accumulation, autophagosome formation, diminished mitochondrial vacuolisation, and protection of vascular basement membrane–matrix interface integrity. Mitochondrial cristae fragmentation may lead to energy metabolism dysfunction, thereby impairing vascular endothelial cell function. Concurrently, the widening of extracellular matrix spaces could compromise the blood–brain barrier integrity, potentially promoting the progression of CSVD. These findings demonstrate that DQJD exerts neurovascular protective effects at least partially through rescuing the mitochondrial damage. RNA sequencing revealed significant alterations in the PPAR pathway in the CSVD mouse model. The biological functions of PPARs depend on co‐activation by peroxisome proliferator activated receptor gamma coactivator‐1α (PGC‐1α), a critical transcriptional coactivator central to regulating mitochondrial function and antioxidant production [49]. PGC‐1α co‐activates the transcription of nuclear respiratory factors 1 and 2, which subsequently regulate mitochondrial transcription factor A. Mitochondrial transcription factor A translocates to the mitochondrial matrix, stimulating mitochondrial DNA replication and mitochondrial gene expression [50]. Thus, in this experimental model, we propose that CCH downregulates the PPAR/PGC‐1α axis, leading to mitochondrial damage. DQJD may stimulate mitochondrial biogenesis by activating the PPAR pathway to upregulate PGC‐1α expression.

Selective targeting of neuroinflammation is considered to be a crucial segment in the treatment of cerebrovascular and neurodegenerative diseases [51]. Therefore, improving neuroinflammation is considered a promising strategy for the treatment of CSVD. Our data suggested that CCH significantly increased the release of TNF‐α, COX‐2, and DQJD treatment significantly decreased the level of TNF‐α. Recently, CCH was demonstrated to activate the inflammasome signalling pathways, involving NLRP3 and AIM2 inflammasomes that critically regulate IL‐1β production [52]. Several recent studies have demonstrated that DQJD exerts neuroprotective effects against cerebral ischaemia–reperfusion injury through the suppression of inflammatory responses. In a clinical study conducted by Chao et al. [8], the levels of IL‐8, IL‐6 and TNF‐α in the serum of patients treated with basic Western medicine in combination with DQJD had an obvious downregulation versus the control patients, which facilitated the recovery from acute cerebral infarction. DQJD significantly reduced TNF‐α levels while exhibiting no significant effects on COX‐2 or ICAM‐1 expression, suggesting a selective mechanism of action. Therefore, we cannot exclude that DQJD can regulate the inflammatory process through other pathways. For example, we can learn from Figure 5A that one of the GOBP processes was significantly enriched after treatment with DQJD compared to the model group (negative regulation of inflammatory response, containing genes such as Adora2a, Pbk). Under CCH‐induced hypoxic conditions, astrocytes are activated and subsequently provoke inflammatory responses by releasing pro‐inflammatory cytokines (IL‐1β, TNF‐α and IL‐6). Conversely, Adora2a activation suppresses astrocyte‐mediated cytokine secretion through the STAT3/YKL‐40 signalling axis [53]. Therefore, we suggest that DQJD may suppress cytokine secretion and inflammatory responses through activating Adora2a. Further investigation is needed to clarify this point in the future.

To investigate the mechanism of DQJD at the transcriptional level, RNA‐seq analysis on the cerebral cortex was conducted. A total of 20 DEGs (12 up‐regulated and 8 down‐regulated) were screened in the model versus the control group. DQJD treatment resulted in 46 DEGs (44 up‐regulated and 2 down‐regulated) versus the model group. Notably, unique gene expression patterns emerged in the cortex of the model group versus the control rats, including those related to immune function (e.g., *Per1, Arl5b, Nr4a3, Per2, Postn, Sstr2, Pcdhb6, Shisa8, Lox, Carpt, Kmo, Capn6 and Gng11*), vascular function (*Pcdhb5, Pcdhga2 and Pcdhb8*) and cytoskeleton (*Col23a1, Pcp4 and Gask1b*) related genes (Figure 4E). Additionally, significant upregulation of the PPAR pathway is observed in the DQJD‐treated CCH model. PPAR, a nuclear hormone receptor, controls genes participating in energy metabolism and inflammation [54]. Increasingly, PPAR‐γ (one of the three isoforms of PPAR) has been confirmed to exert an essential part in angiogenesis in the brain [55]. It stimulates the synthesis and secretion of angiogenic factors [56, 57]. For example, Kernan et al. demonstrated that pioglitazone, a PPAR‐γ agonist, prevented stroke or myocardial infarction in 3 out of 100 diabetic patients during a 5‐year observation period [58]. Studies have shown that in the setting of ischaemic injury, increased mRNA and protein expression of PPAR‐γ was detected in both neurons and microglia [59]. The highest level was detected 24 h after ischaemia, and PPAR‐γ protein levels were still significantly elevated at 14 days [60]. Collectively, PPAR‐γ activation has demonstrated therapeutic potential in CSVD.

In the present study, we utilised a network pharmacology approach to predict the active components and intrinsic mechanisms of DQJD in the therapy for CSVD. Based on network pharmacology, 104 hub compounds and 54 core target genes were identified. The possible main ingredients of DQJD in treating CSVD were bis[(2S)‐2‐ethylhexyl] benzene‐1,2‐dicarboxylate, dehydroeburicoic acid, glyasperin F and licochalcone G. AKT1, IL6, TNF, and ALB were identified as hub targets. In the anti‐inflammatory test, dehydroeburicoic acid significantly decreased the TNF‐α and IL‐1β levels in the serum of mice [61]. Glyasperin F may mediate cell growth by inhibiting the activation of MMP1, thus reducing cell death and tissue damage caused by the inflammatory response [62]. Licochalcone G is a flavonoid with antioxidant effects and can protect nerve cells and cognitive function [63]. AKT1, a subtype of serine/threonine kinase, is essential for inducing cell division, preventing apoptosis and promoting angiogenesis. AKT1 knockdown improves hippocampal neurogenesis and learning and memory deficits in phosphatase and tensin homologue mutant mice [64]. TNF‐α secreted by perivascular M1‐like microglia induces endothelial necroptosis, contributing to BBB damage. Anti‐TNFα treatment notably alleviates BBB disruption and relieves stroke‐induced cerebrovascular injury [65]. The enrichment analysis results suggest that the underlying mechanism of DQJD in the treatment of CSVD involves the VEGF signalling pathway. VEGF‐α is a growth factor with angiogenic activity and a neurotrophic factor with protective effects on neural cells [40]. It promotes vascular endothelial cell development and proliferation and has been shown to enhance synaptic plasticity, which is beneficial in experimental stroke. Dysregulation of VEGF is pathologically linked to ischaemic cerebrovascular disorders, such as vascular dementia, stroke and chronic cerebral ischaemia, through mechanisms involving blood–brain barrier disruption, neuroinflammation and impaired vascular homeostasis [66]. The upregulation of VEGF‐α expression promotes the proliferation of neural stem cells, thereby reducing neuronal damage in ischaemic rats and improving neurobehavioral functions [41]. Administration of VEGF‐α reduced the infarct volume in rats with ischaemia–reperfusion brain injury [67]. In conclusion, VEGF‐α plays a multifaceted role in cerebral ischaemia–reperfusion diseases, including the promotion of angiogenesis and neuroprotection, suggesting that VEGF‐α may be a potential therapeutic target for ischaemic brain diseases.

Integrated transcriptomics and network pharmacology analysis demonstrated that the therapeutic effect of DQJD on the CCH model might be mediated by the activation of the RXR‐γ/PPAR‐γ/VEGF‐α signalling pathway. PPAR‐γ, one of the three isoforms of PPAR, stimulates the synthesis and secretion of angiogenic factors, thus has been confirmed to exert an essential part in angiogenesis in the brain. Cerebral ischaemia reduces PPAR‐γ DNA‐binding activity, thereby disrupting its transcriptional regulation of target genes (e.g., VEGF) and ultimately exacerbating neuroinflammation and neuronal damage [68]. PPAR‐γ P465L mutant mice possess only half of the PPAR‐γ activity compared to wild‐type control [69]. Infarct volumes induced by transient focal ischaemia in PPAR‐γ P465L mutant mice were twice as large as their wild‐type litter mates control [70]. Conditional neuron‐specific PPAR‐γ knockout mice exhibited significantly increased cerebral damage and oxidative stress following middle cerebral artery occlusion [71]. Systemic and lateral ventricular application of thiazolidinedione (PPAR‐γ agonist) after transient occlusion of the middle cerebral artery or common carotid artery reduces COX‐2 expression in the peri‐infarct cortical area [72]. Recent studies have shown that the upregulation of PPAR‐γ enhances the activity of ICAM‐1, matrix metalloproteinase‐9, and several inflammatory mediators in the ischaemic brain area [73]. Studies have shown that PPAR‐γ agonist can still reduce infarct size and restore some neurological functions through suppressing inflammatory mediators (IL‐1β, COX‐2 and iNOS) 22 days after the intervention, suggesting that PPAR‐γ is an endogenous protective target [74]. Similarly, studies have shown that PPAR‐γ and its agonist ligands have a protective effect against ischaemia–reperfusion injury through the suppression of the inflammatory mediators, including TNF‐α, IL‐1β, ICAM‐1 and COX‐2 [75]. Furthermore, the biological function of PPAR‐γ is dependent on its heterodimerised form, a complex of PPAR‐γ‐RXR‐γ. After activation by the PPAR activator or ligand, the PPAR‐RXR transcriptional complex recruits the co‐activator factor and subsequently binds to the PPAR response element to regulate gene transcription, including the inflammatory and vascular response of endothelial and vascular smooth muscle cells [76]. The transcriptional process can be activated only after RXR‐γ binds to PPAR‐γ, which in turn regulates the expression of VEGF [42]. There is evidence that 15‐deoxy‐Δ (12, 14)‐prostaglandin J2 (PPAR‐γ ligand) and ginkgolic acid (PPAR‐γ agonist) could both induce increased VEGF expression. Activation of T cell receptors blocked the IL‐10, IL‐9, IL‐13 and VEGF‐α expression and production [77]. VEGF‐α (a growth factor with angiogenic activity) promotes vascular endothelial cell development and proliferation. VEGF‐α upregulation stimulates neural stem cell proliferation through angiogenic signalling pathways, mitigating cerebral ischaemia‐induced neuronal damage and ameliorating neurobehavioral deficits in rodent models [61]. Shen et al. [78] demonstrated that VEGF enhanced the ability of reactive astrocytes to reprogram into new neurons in the adult brain after ischaemic injury. Our results demonstrated that CCH decreased the levels of RXR‐γ, PPAR‐γ and VEGF‐α, while DQJD treatment reversed the levels of all three proteins. This suggests that DQJQ may alleviate inflammatory injury and promote angiogenesis by upregulating RXR‐γ, PPAR‐γ and VEGF‐α levels. The question of whether DQJD can promote the conversion of astrocytes into neurons deserves further exploration.

**FIGURE 8:**
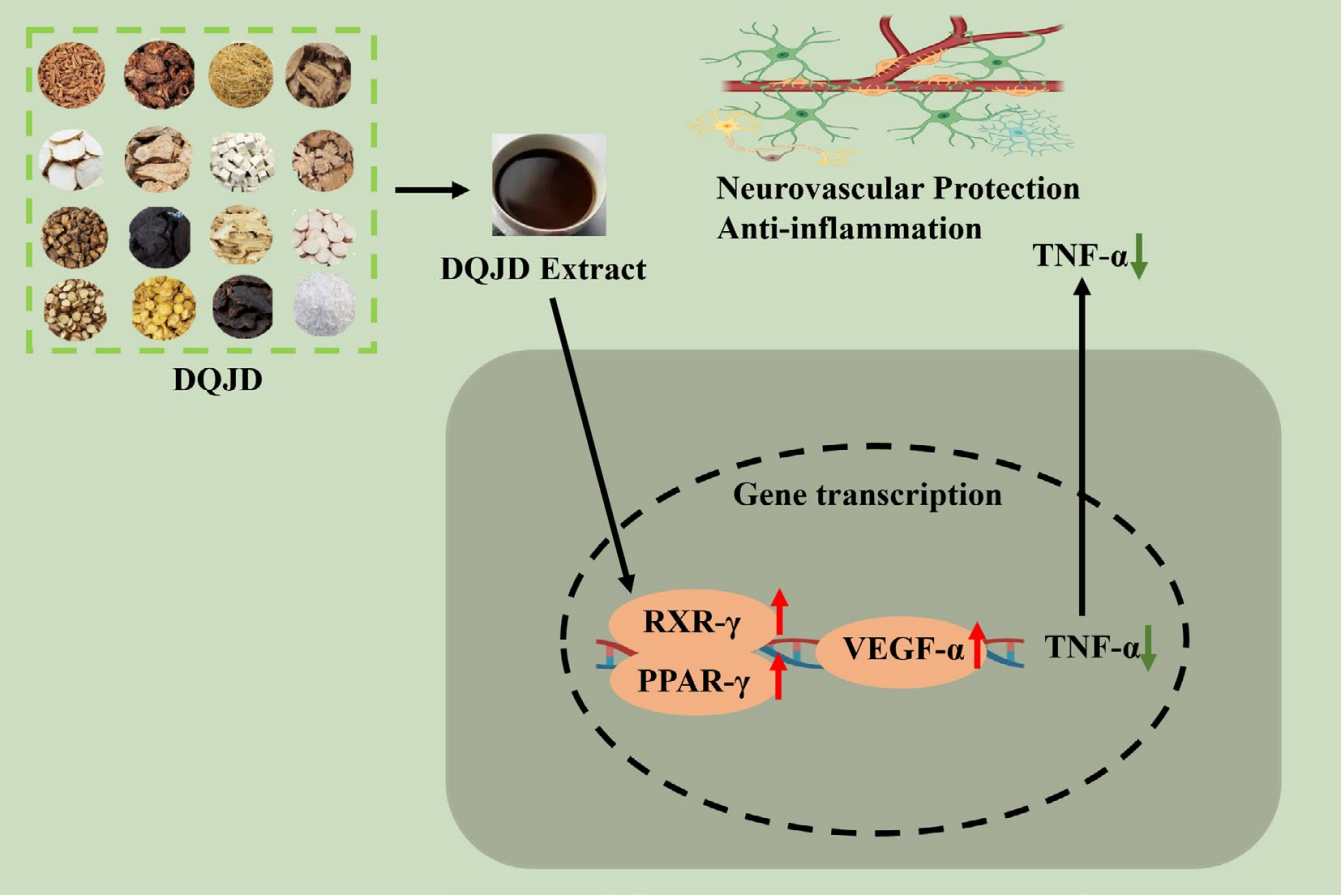
The possible mechanism of DQJD on CSVD. DQJD has beneficial effects on improving inflammation and pathological damage in the cortex and hippocampus in the CSVD model. The mechanism of action may be related to the activation of the RXR‐γ/PPAR‐γ/VEGF signalling pathway.

In conclusion, the present study demonstrated that DQJD may inhibit the inflammatory response, exert neurovascular protection and improve cognitive function in CCH rats through the RXR‐γ/PPAR‐γ/VEGF‐α signalling pathway (Figure 8) and multiple targets (RXRG, AKT1 and TNF). Mandenol, dehydroeburicoic acid, glyasperin F and licochalcone G may be potential active components of CSVD. However, our experimental studies on the mechanism of DQJD against CCH‐induced rats only revealed the tip of the iceberg. We will pursue more in‐depth mechanistic studies based on transcriptomics, for example, whether DQJD has better immunomodulatory effects on microglia cells or astrocytes. In addition, we have to figure out the chemical basis of DQJD in the future to fully explain the pharmacodynamic mechanism. Additional validation experiments with pathway inhibitors or knockout mice are needed to confirm how DQJD protects the cerebral vasculature. Finally, based on the results from the network pharmacology analysis, the PI3K‐AKT pathway exhibited the highest enrichment, suggesting it as a potential additional mechanism through which DQJD exerts its therapeutic effects. The PI3K/AKT signalling pathway regulates neurotoxicity and facilitates neuronal survival through different substrates, including mTOR, FOXOs, GSK‐3β, and caspase‐9. A current study demonstrated that the toll‐like receptor (TLR2 and TLR4) antagonist activates the PI3K/Akt/GSK3β signalling pathway to inhibit neuronal apoptosis and reduce pro‐inflammatory factor expression, thereby exerting protective effects against CSVD [79]. Similarly, activation of the PI3K/Akt pathway has been demonstrated to downregulate Bax and Caspase‐3 levels, thereby inhibiting neuronal apoptosis and ameliorating the cellular morphology of brain tissue and cognitive functions in rats [80]. Therefore, whether PI3K‐AKT is one of the mechanisms underlying DAJD's therapeutic effects against CSVD warrants further investigation.

## 5 Conclusion

Our study confirmed that DQJD displayed an anti‐CSVD effect. DQJD ameliorated damages in cortical and hippocampal neurons and reduced neuroinflammation in the classical CCH surgery model rats. Transcriptome sequencing and network pharmacology were first used to identify potential targets and mechanisms. As a result, DQJD exerts neurovascular protective effects by activating the RXR‐γ/PPAR‐γ/VEGF‐α pathway. This study shows the application potential of DQJD in the therapy for CSVD and provides theoretical support for its clinical application in the precise treatment of neurovascular injury.

## Author Contributions

**Mengna Lv:** formal analysis (equal), investigation (equal), writing – original draft (equal). **Xiaolu Yang:** writing – review and editing (equal). **Xiaolu Shi:** investigation (equal), visualization (equal), writing – original draft (equal). **Shengxuan Cao:** investigation (equal), visualization (equal). **Wenjie Li:** visualization (equal), writing – original draft (equal). **Mingmei Zhou:** conceptualization (equal), methodology (equal), writing – review and editing (equal). **Xiaojun Gou:** data curation (equal), formal analysis (equal). **Ying Huang:** conceptualization (equal), methodology (equal), writing – review and editing (equal).

## Disclosure

Standardisation statement: To ensure pharmacological consistency, DQJD preparation followed with three‐tier quality control: (1) All herbs of DQJD were purchased from Beijing Tong Ren Tang Co. Ltd. (Table 1), and authenticated by Dr. Xirong He from the China Academy of Chinese Medical Sciences (Beijing, China). All herbs comply with the property regulations under the relevant items of the 2020 edition of the Chinese Pharmacopoeia; (2) Critical processing parameters were strictly controlled. Soak herbs in water for 30 min. After boiling with high heat, continue to decoct for 20 min to obtain a water decoction. Add water to the medicinal residues and continue to decoct for 20 min. Combine 2 times the water decoction and concentrate to 1 mg/mL crude drug, refrigerated for later use; (3) Chromatographic fingerprint analysis (HPLC, Figure S1).

## Conflicts of Interest

The authors declare no conflicts of interest.

## Supporting information


Appendix S1

## Data Availability

The datasets used and/or analysed during the current study are available from the corresponding author on reasonable request.
