# Supplementary material for: Daqinjiao Decoction Ameliorates CSVD via RXR‐γ/PPAR‐γ/VEGF‐α Pathway: Insights From Transcriptome Sequencing and Network Pharmacology
Source: J Cell Mol Med. 2025 Jul 19;29(14):e70712. doi: 10.1111/jcmm.70712 (PMC12274961; doi:10.1111/jcmm.70712)
Supplement: Supplementary file 1 — Appendix S1 [file JCMM-29-e70712-s001.docx]

**Supplementary Materials**

**Daqinjiao decoction ameliorates CSVD via RXR-γ/PPAR-γ/VEGF-α pathway: Insights from transcriptome sequencing and network pharmacology**

Mengna Lv ^1, 2 †^, Xiaolu Yang ^3, †^, Xiaolu Shi ^2^, Shengxuan Cao ^2^，Wenjie Li ^2^, Mingmei Zhou ^1, *^, Xiaojun Gou ^3, *^, Ying Huang ^2, *^

1. **Daqinjiao Decoction Quality Control**

The method of extracting Daqinjiao decoction (DQJD) in this study was carried out according to the method of Li et al., (Ref18 in the manuscript) with modifications. According to the method of Li et al., the herbal mixture was pulverized to a coarse powder (24-mesh sieve, ISO 3310-1 standard), then soaked in 1000 mL distilled water for 10 min. The herbs were decocted in pouches using high-intensity heating (200 ℃) until boiling, followed by low-intensity heating (80 ℃) until the liquid volume reduced to 80% of the original (≈ 800 mL). Post-decoction, it was vacuum-dried and ground to obtain the dry powder. We optimized the existing methodology based on his approach. Firstly, the soaking duration was prolonged from 10 to 30 minutes. Additionally, after boiling with high heat, continue to decoct for 20 minutes to obtain a water decoction. Add water to the medicinal residues and continue to decoct for 20 minutes. Combine 2 times of water decoction and concentrate to 1 mg/mL crude drug, refrigerated for later use.

Weigh a certain weight of decoction pieces (Table 1) and soak them in water for 30 minutes. After boiling with high heat, continue to decoct for 20 minutes to obtain a water decoction. Add water to the medicinal residues and continue to decoct for 20 minutes. Combine 2 times of water decoction and concentrate to 1 mg/mL crude drug, refrigerated for later use. The results show that the HPLC method for content determination is simple, reliable, and replicable and can be used for the quality control of DQJD.

**1.1 Instruments and reagents**

Waters E2695 high performance liquid chromatograph, acetonitrile (chromatographically pure, purchased from Merck, Germany); ultrapure water. *Gentianae Macrophyllae Radix* (Lot.20201004), *Chuanxiong Rhizoma* (Lot. 20091303), *Angelicae Pubescentis Radix* (Lot.200828001), *Angelicae Sinensis Radix* (Lot.21031501), *Paeoniae Radix Alba* (Lot.21040902), *Gypsum Fibrosum* (Lot.190724002), *Glycyrrhizae Radix et Rhizoma* (Lot.201121004), *Notopterygii Rhizoma et Radix* (Lot.19121302), *Saposhnikoviae Radix* (Lot.20101501), *Angelicae Dahuricae Radix* (Lot.200825001), *Scutellariae Radix* (Lot.20061402), *Atractylodis Macrocephalae Rhizoma* (Lot.21041901), *Poria* (Lot.200948), *Rehmanniae Radix* (Lot.20122303), *Rehmanniae Radix Praeparata* (Lot.200827), *Asari Radix et Rhizoma* (Lot.200929001) were purchased from Beijing Tongrentang. All herbs of DQJD were purchased from Beijing Tong Ren Tang Co., Ltd., and authenticated by Dr. Xirong He from the China Academy of Chinese Medical Sciences (Beijing, China). All herbs comply with the property regulations under the relevant items of the 2020 edition of the Chinese Pharmacopoeia.

Loganic acid (Lot.P01A9L67130), gentiopicroside (Lot.Y30J9Q66926), paeoniflorin (Lot.M28GB143089), cimicifugin (Lot.Y03J11H117538), ferulic acid (Lot.G27A11L112005), glycyrrhizin (Lot.Z07J12X136344), 5-o-methylvisalaitol glycoside (Lot.Y24J11H119521), baicalin (Lot.Z28S11X125952) and ammonium glycyrrhizinate (Lot.M04GB140062) were purchased from Yuanye Bio-Technology Co., Ltd (purity > 98%, Shanghai, China).

**1.2. Chromatographic conditions**

Chromatographic column: Agilent 5 TC-C18 (250 mm×4.6 mm, 5 μm); mobile phase: 0.1% phosphoric acid aqueous solution (A)-acetonitrile (B); detection wavelength 237 nm; flow rate: 1.0 mL/min; injection volume 10 μL; column temperature: 30 °C; elution program: 0-5-10-22-30-35-42-53-60-90 min, 5-10-10-12-13-13-22-27-30 -80 B (%).

**1.3. Extraction of DQJD samples**

Weigh 9 g of Qinjiao, 6 g of Gancao, Chuanxiong, Danggui, Baishao, Shigao and Duhuo, 3 g of Qianghuo, Fangfeng, Huangqin, Baizhi, Baizhu, Shengdihuang, Shudihuang and Fuling, 1.5 g of Xixin into a 1000 mL round bottom flask, add 800 mL of water, soak for 10 minutes, and extract under reflux for 30 minutes. The extract was collected, heated and concentrated to 1 g crude drug/mL (80 mL), freeze-dried to obtain DQJD freeze-dried powder, which was stored at 4°C for later use.

**1.4. Preparation of Test Sample Solutions**

Take 0.5 g of freeze-dried powder and put it in a conical flask, add 30 mL of methanol, sonicate for 20 min, mix well, filter with a 0.45 μm organic filter membrane, and take the subsequent filtrate as the test solution.

**1.5. Preparation of reference material stock solutions**

Precisely weighed and accurately weighed loganic acid 5.10 mg, gentiopicroside 4.81 mg, paeoniflorin 5.22 mg, cimicifugin 4.92 mg, ferulic acid 4.93 mg, glycyrrhizin 5.18 mg, 5-o-methylvisalaitol glycoside 5.09 mg, ammonium glycyrrhizinate 5.06 mg, respectively dissolved in 5 mL Methanol was prepared into reference solution of 1.02 mg/mL, 0.962 mg/mL, 1.044 mg/mL, 0.984 mg/mL, 0.986 mg/mL, 1.036 mg/mL, 1.018 mg/mL, 1.012 mg/mL, and accurately weighed. 5.61 mg of baicalin was dissolved in 10 mL of methanol to prepare a reference solution of 0.561 mg/mL. Take 120 μL of loganic acid, 400 μl of gentiopicroside, 60 μL of paeoniflorin, 20 μL of cimicifugin, 20μL of ferulic acid, 20 μL of glycyrrhizin, 10 μL of 5-o-methylvisalaitol glycoside, 150 μL of baicalin, and 30 μL of ammonium glycyrrhizinate, respectively, and add methanol to 2 mL to obtain a mixed reference substance solution.

**1.6. Measurement method**

Accurately draw 10 μL of the reference solution and the test solution and inject them into the liquid chromatography for determination.

**1.7. Results**

The chromatographic separation of loganic acid, gentiopicroside, paeoniflorin, cimicifugin, ferulic acid, glycyrrhizin, 5-o-methylvisalaitol glycoside, ammonium glycyrrhizinate, and baicalin is good, and the results are shown in **Figure S1A**. The chromatographic separation of each reference in the test sample is good, and the results are shown in **Figure S1B**. The HPLC chromatograms and test grade blank methanol solution are shown in **Figure S1C**.


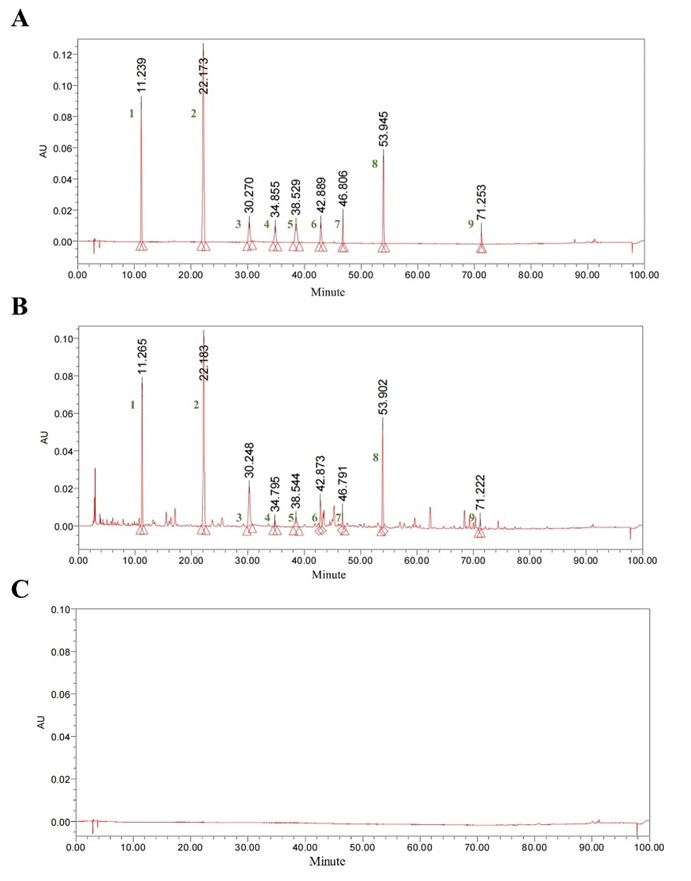


**Figure S1. Representative HPLC chromatograms of mixed standards, DQJD sample and, blank methanol solution at 237 nm.** (A) mixed chemical standards. (B) DQJD sample. (C) blank methanol solution. 1. Loganic acid (retention time: 11.239); 2. Gentiopicroside (retention time: 22.173); 3. Paeoniflorin (retention time: 30.270); 4. Cimicifugin (retention time: 34.855); 5. Ferulic acid (retention time: 38.539); 6. Glycyrrhizin (retention time: 42.873); 7. 5-o-methylvisalaitol glycoside (retention time: 46.791); 8. Baicalin (retention time: 53.945); 9. Ammonium glycyrrhizinate (retention time:71.253).**2.** **Neurobehavioral experiments**

The behavioral assessments were conducted 12 weeks after DQJD administration. The experimental procedures were performed by investigators blinded to group allocation throughout data collection and analysis phases.

**2.1 Instruments**

General Morris Water Maze Video Analysis System for Rats and Mice (Shanghai, China, Model: JLBehv-MWMG), and General Spontaneous Activity Video Analysis System.

**2.2 Open field test**

The rats in each group were put into the center of the bottom surface of the open field box, and were allowed to explore freely. The total movement scores of the animals were observed and recorded within 5 min, including horizontal exercise scores (horizontally crossing grids) and vertical movement scores (number of rearing instances). After test, disinfection was performed with alcohol spray to remove the residual odor of the rats and clean the excrement in the open field box.

- 1. **Morris water maze evaluation of learning and memory ability of rats**

The bucket was filled with water to a predetermined level, followed by the addition of carbon black ink to make the water surface cloudy and opaque. The water temperature in the bucket was heated to 25±2 °C. The pool was divided into four quadrants equally, and the platform was fixed at the center of the third quadrant. The platform was immersed 3 cm below the liquid level of the pool, and there were the fourth, first and second quadrants, respectively in a clockwise direction.

**Training Phase:** During training, the rats were placed on the platform for 20 seconds, and then the rat's head were gently placed into the pool from the first, second, third, and fourth quadrants to allow them to find their own platform position. The training was conducted once a day. Rats that remained on the platform for 3 s were considered successful on the platform. If the subjects failed to reach the platform within the 90-second time limit, they were guided to remain on it for 20 seconds before proceeding to the next quadrant test. The escape latency was recorded as the maximum allotted duration (90 s).

**Positioning and navigation phase:** Days 1 to 3 were the positioning and navigation phase. The arithmetic mean of 4 latent periods per day was used as the result of this day, and the escape latency of each group was calculated and compared.

**Space exploration phase:** The fourth day was the space exploration stage. When the platform was removed, the rats were put into the water from the original 4 positions. The number of times the rats crossed the original platform area and the cruising distance within 90s were recorded.

**2.4 Statistical analysis**

Statistical data were analyzed using GraphPad Prism version 8 software. Experimental data were expressed as mean ± standard deviation (SD). Shapiro-Wilk test was performed to assess the normality of the data distribution and p- value ≥ 0.05 means closely to a normal distribution. One-way analysis of variance (ANOVA) followed by Tukey's post hoc test were utilized to analyze the statistically significant differences among multiple groups. Statistical significance was defined as *p* < 0.05.

**2.5 Results**

**2.5.1 Open field test**

As shown in **Figure S2** and **Table S1,** compared with the control and sham groups, the model group had a longer stay time on the four sides of the open field box, an increasing trend in the total movement distance, and a decrease in the number of activities, but there was no significant difference. Compared with the model group, DQJD group had significantly shortened total movement distance (p<0.05), and significantly increased of activities frequency (p<0.05).

**
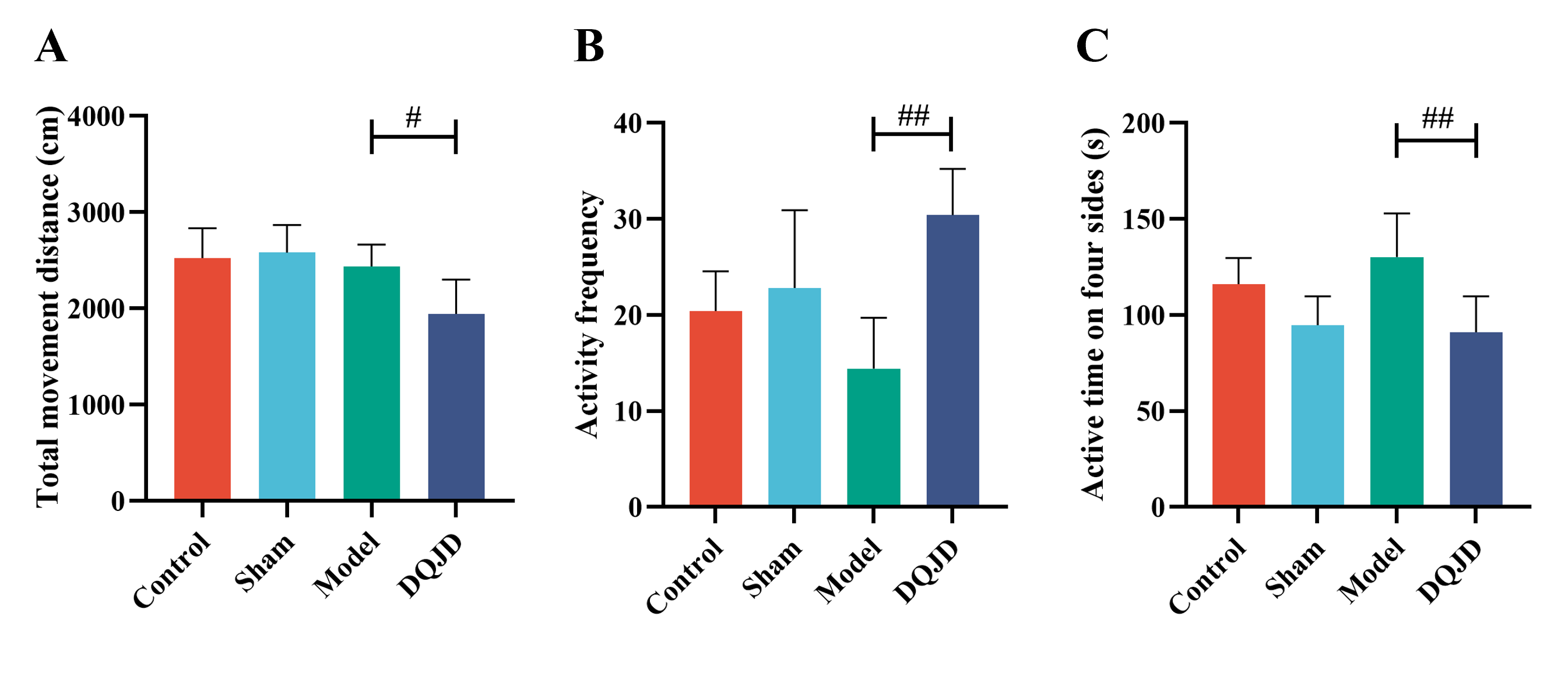
**

**Figure S2. DQJD improved the results of OFT.** (A) Total movement distance (cm). (B) Activity frequency. (C) Active time on four sides (s). Values are expressed as mean ± SD (n = 5). ^#^ *P* <0.05 (versus Model), ^##^ *P* <0.01 (versus Model).

**Table S1. Open field test results of rats in each group (‾x±s) (n=5)**.

| Group | Total movement distance (cm) | Activity frequency | Active time on four sides (s) |
| --- | --- | --- | --- |
| Control | 2424.09±371.93 | 18.5±5.96 | 119.88±15.18 |
| Sham | 2473.7±368.41 | 20.83±8.7 | 98.64±16.57 |
| Model | 2546.29±335.45 | 13.17±5.64 | 124.77±24.36 |
| DQJD | 1878.23±358.78^#^ | 27.5±8.31^#^ | 87.38±18.97^*^ |

**p*<0.05 vs. Control group; ^#^*p*<0.05 vs. Model group.

**2.5.2 Morris water maze test**

In the model group, the escape latency was significantly prolonged on days 1 and 2, and there was still a trend of prolongation on day 3. Compared with the model group, the escape latency had a tendency to shorten on days 1, 2, and 3 in the DQJD group with no significant difference (**Figure S3A-C** and **Table S2**). Compared with the control group, the model group showed a downward trend in the proportion of target quadrant residence time (p<0.05). Compared with the model group, the proportion of stay time in the target quadrant had an upward trend in DQJD group (**Figure S3D** and **Table S2**).

**
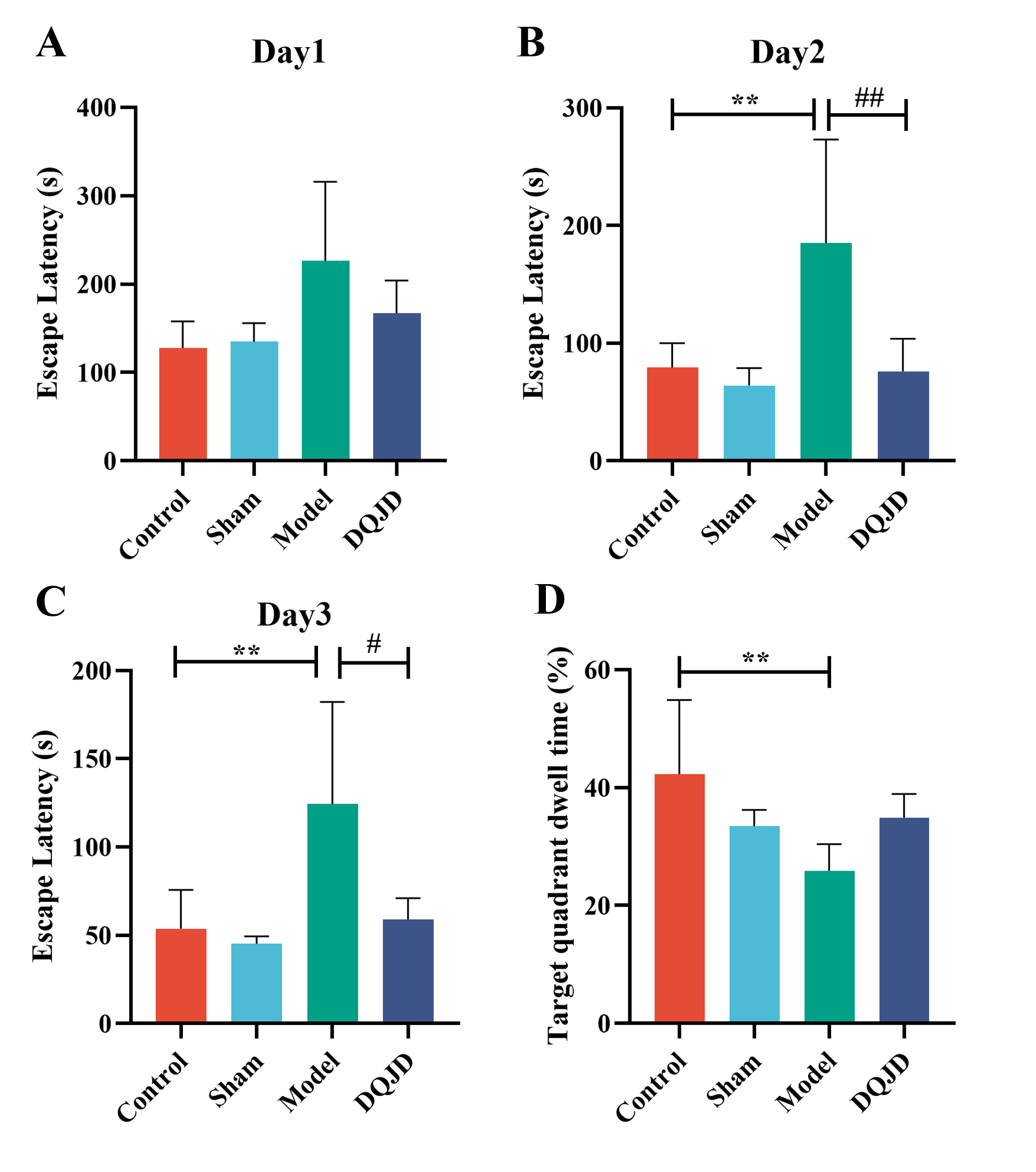
**

**Figure S3. DQJD improved the results of** **Morris water maze test.** (A) Escape Latency (s) in day1. (B) Escape Latency (s) in day 2. (C) Escape Latency (s) in day3. (D) Target quadrant dwell time (%). Values are expressed as mean ± SD (n = 5). * *P* <0.05 (versus Control), ** *P* <0.01 (versus Control); ^#^ *P* <0.05 (versus Model), ^##^ *P* <0.01 (versus Model).

**Table S2. Morris water maze test results of rats in each group (‾x±s) (n=5).**

| Group | Escape Latency (s) | | | Target quadrant dwell time (%) |
| --- | --- | --- | --- | --- |
|  | day 1 | day 2 | day 3 |  |
| Control | 133.67±30.41 | 87.67±27.42 | 59.33±23.86 | 39.62±13.01 |
| Sham | 152±46.14 | 72±23.69 | 46.33±4.68 | 32.18±4.02 |
| Model | 225.67±79.96^*^ | 171.67±85.24^*^ | 113±58.81 | 27.33±5.31^*^ |
| DQJD | 179.67±45.43 | 85.33±33.36^#^ | 72.83±35.54 | 33.58±4.88 |

**p*<0.05 vs. Control group; #*p*<0.05 vs. Model group.
